# Quantum Mechanics/Molecular Mechanics Simulations Distinguish Insulin-Regulated Aminopeptidase Substrate (Oxytocin) and Inhibitor (Angiotensin IV) and Reveal Determinants of Activity and Inhibition

**DOI:** 10.1021/acs.jcim.5c00869

**Published:** 2025-06-11

**Authors:** Marko Hanževački, Rebecca M. Twidale, Eric J.M. Lang, Will Gerrard, David W. Wright, Vid Stojevic, Adrian J. Mulholland

**Affiliations:** ‡ Centre for Computational Chemistry, School of Chemistry, 1980 University of Bristol, Bristol BS8 1TS, United Kingdom; § Kuano, Hauxton House, Mill Scitech Park, Mill Lane, Cambridge CB22 5HX, United Kingdom

## Abstract

Insulin-regulated aminopeptidase (IRAP) is a zinc-dependent metalloenzyme identified as a novel target for combating diabetes-induced diseases due to its crucial role in glucose metabolism and insulin sensitivity regulation. IRAP’s catalytic domain catalyzes the N-terminal peptide bond hydrolysis of natural substrate oxytocin, a neuroactive peptide linked to improved cognition and other elemental brain functions. Angiotensin IV and similar peptides are recognized as cognitive enhancers due to their ability to competitively inhibit IRAP’s proteolytic activity, thereby mitigating natural neuropeptide degradation. Despite a very similar binding complex between the substrate and the inhibitor with IRAP, particularly around the scissile bond, it is unclear why the enzyme metabolizes oxytocin but does not efficiently degrade angiotensin IV. We employed enhanced sampling quantum mechanics/molecular mechanics (QM/MM) molecular dynamics simulations and higher-level QM/MM calculations to explore the reaction of these two peptides in IRAP. The calculated energy barrier for oxytocin cleavage was in very good agreement with the experimental data. A significantly higher energy barrier for the formation of the oxyanion tetrahedral intermediate (TI) and a higher overall barrier for the peptide cleavage were observed for the reaction with angiotensin IV. Comprehensive electronic structure analysis utilizing NBO and NCI methods unveiled the molecular basis for different reactivity, a stabilizing interaction between the sigma hole of the N-terminus disulfide bond and the hybridizing lone pair of the scissile peptide nitrogen in oxytocin. The interplay between a weak noncovalent spodium bond and strong bidentate coordination of the catalytic Zn^2+^ by angiotensin IV caused a larger deviation of the valine C–Cα-Cβ angle from ideal tetrahedral geometry, consequently destabilizing the TI. These results underscore the critical importance of analyzing the dynamics, interactions, and electronic properties of reaction intermediates and transition states in enzymatic processes. Our findings have significant implications for the rational design and development of IRAP inhibitors as potential therapeutic agents for memory disorders, neurodegenerative diseases, and diabetes.

## Graphical abstract

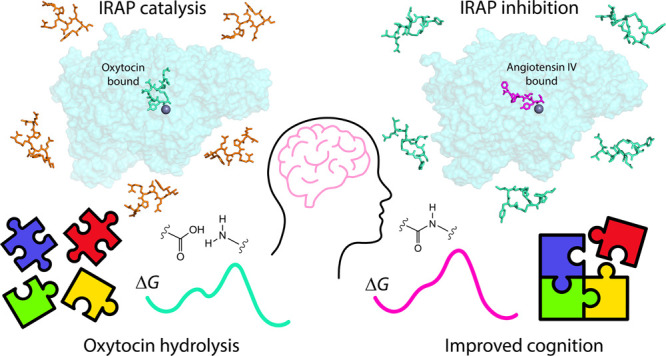

## Introduction

Insulin-regulated aminopeptidase (IRAP) is a type II transmembrane protein with its C-terminal catalytic domain located at the cell exterior.^1–5^ IRAP is found in almost all human tissues and is known to be expressed in neuronal cells, placental cells, and leukocytes.^6^ IRAP belongs to the family of zinc-dependent M1 metallopeptidases,^7^ which includes structurally analogous human aminopeptidase N (APN),^8,9^ aminopeptidase A (APA),^10^ and mammalian endoplasmic reticulum aminopeptidases 1 (ERAP1)^11,12^ and ERAP2.^13^ IRAP plays diverse biological roles spanning various cellular functions, including human hormone and glucose regulation, and cognitive function.^14^ The crystal structure of IRAP is shown in Figure 1a. Our focus on the catalytic domain of IRAP is guided by the availability of high-resolution structural data, enabling detailed analysis of peptide binding and inhibition. Although the cytoplasmic N-terminal domain is important for trafficking and signaling, its unresolved structure means that it cannot reliably be included in the mechanistic study here. We use experimentally determined structures only here.

**Figure 1:**
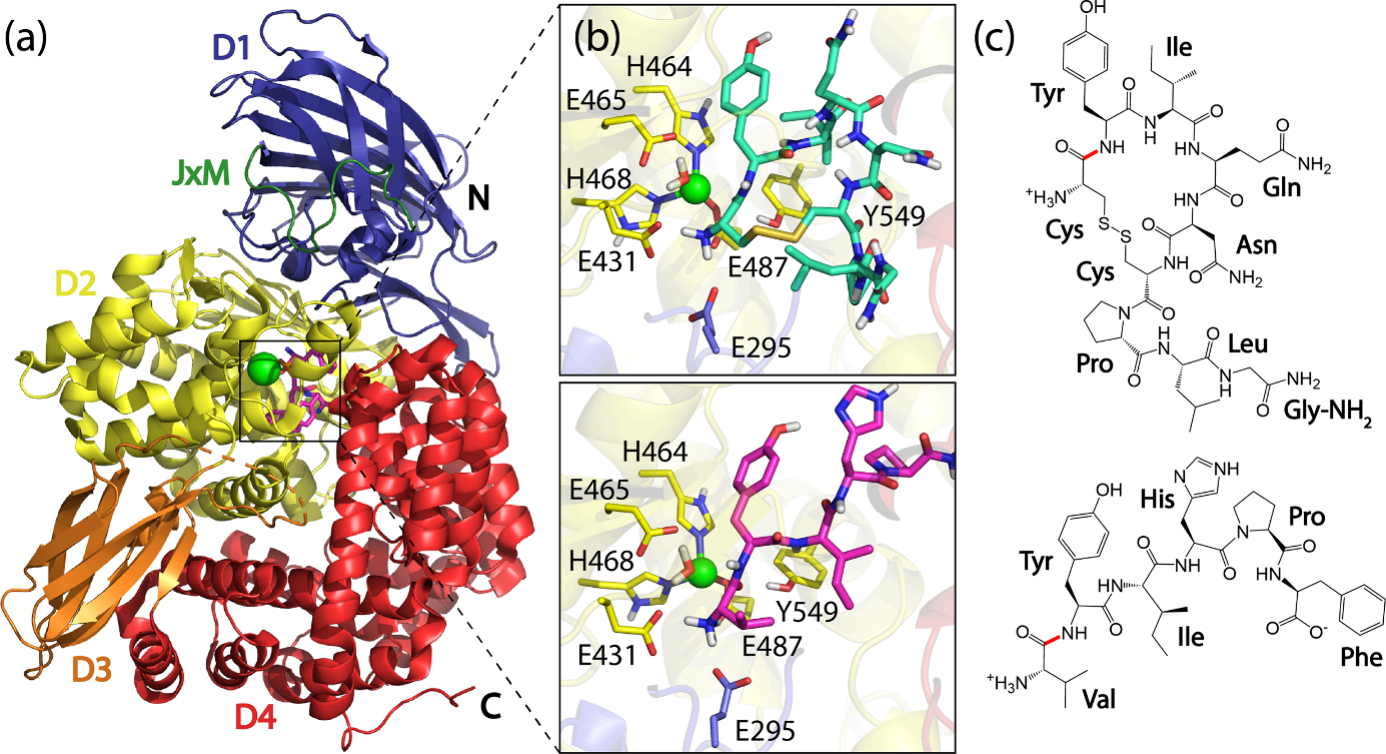
(a) Crystal structure of the catalytic domain of the IRAP monomer in the closed conformation with a phosphinic derivative inhibitor bound near the zinc ion in the active site (PDB ID 5MJ6).^60^ Part of the juxtamembrane (JxM) region, and four domains D1–D4, are shown in different colors, the Zn^2+^ ion is shown as a green sphere, and the inhibitor is shown in magenta stick representation. All other cocrystallized species, water molecules and glycosylation is omitted for clarity. (b) Representative structures of the E-R complex of oxytocin (upper) and angiotensin IV (lower) bound in the active site of IRAP from QM/MM MD simulations. All nonpolar hydrogen atoms and the rest of the GAMEN loop was omitted for clarity. (c) Molecular structures of the IRAP substrate oxytocin (upper) and the inhibitor angiotensin IV (lower) with the scissile peptide bond highlighted in red.

As an insulin-responsive transporter, IRAP facilitates the translocation of glucose transporter 4 (GLUT4)^15–17^ to the plasma membrane in adipose tissue and skeletal muscle cells which allows the uptake of glucose from the bloodstream into these tissues, thereby regulating blood glucose levels.^18^ IRAP’s complex interplay with the GLUT4 in hippocampal neurons and other brain regions is crucial for memory-related cognitive functions. Both proteins translocate to the cell surface upon insulin stimulation, facilitating glucose uptake into neurons.^6,19^

IRAP’s involvement in memory processes is multifaceted. As the angiotensin type 4 (AT4) receptor, IRAP inhibition by angiotensin IV can enhance hippocampal memory, potentially by increasing GLUT4-mediated glucose uptake.^6,20^ Additionally, IRAP may regulate GLUT4 compartmentalization and recycling, affecting glucose availability for cognitive processes. In neurodegenerative conditions and type 2 diabetes, where brain hypometabolism and cognitive impairment are common, IRAP’s role in glucose uptake and memory processes makes it a potential therapeutic target.^21^

IRAP catalyzes the hydrolytic cleavage of the N-terminal residue in peptide substrates. Due to its active site plasticity and large binding pocket, it can hydrolyze substrates of various sizes.^3^ Some examples include the small linear peptide Met-enkephalin and large macrocyclic signaling peptides oxytocin and vasopressin.^22^ Oxytocin, a cyclic neuropeptide, is particularly important due to its positive effects on human behavior and social functioning,^23^ as well as its pivotal role in initiating and maintaining uterine contractions during labor.^24^

IRAP has also been identified as the AT4 receptor,^25^ binding the linear hexapeptide angiotensin IV, a metabolite of the hypertensive angiotensin II.^26^ Although the predicted binding mode of angiotensin IV to IRAP is almost identical to that of oxytocin, it is not hydrolyzed by IRAP,^27^ instead, it is a competitive inhibitor, with *K*_i_ in the high nanomolar range. It blocks the proteolytic activity of IRAP and so limits breakdown of natural substrates^28,29^ (see Figure 1b for representative structures of oxytocin and angiotensin IV bound to IRAP, from molecular simulations).

Due to their ability to inhibit IRAP, angiotensin IV peptide analogs and peptidomimetics could be used as scaffolds for development of pharmaceutical leads,^30^ which could potentially increase levels of peptide hormones in the brain and improve cognitive function such as learning and memory in pathological conditions such as Alzheimer’s disease.^31^ Moreover, inhibition of IRAP has been shown to improve insulin sensitivity and glucose uptake in insulin-sensitive tissues, which makes IRAP promising target for the treatment of type 2 diabetes.^28,32^ Therefore, IRAP inhibitors could be developed as cognitive enhancers, and IRAP levels or activity might serve as biomarkers for cognitive decline or altered brain glucose metabolism. Molecular structures of the substrate oxytocin and the inhibitor angiotensin IV are shown in Figure 1 **c.**

The catalytic mechanism of M1 aminopeptidases has been extensively studied,^8,33–36^ yet the molecular basis for IRAP’s differential activity toward peptide substrates like oxytocin and vasopressin compared to hydrolyzable inhibitor angiotensin IV remains elusive. While the majority of metallopeptidase molecular modeling studies have focused on enzymes outside of the M1 family,^37–41^ the hydrolysis mechanism is presumed to be conserved across the class due to the presence of conserved catalytic glutamate and tyrosine residues, as well as analogous Zn^2+^ coordination motifs.^2,42^ In addition to the catalytic glutamate, a conserved tyrosine residue polarizes the enzyme–substrate complex stabilizing the oxyanion tetrahedral intermediate (TI) that forms during the hydrolysis reaction. This mechanism has been demonstrated in related enzymes such as leukotriene A4 hydrolase^43^ and aminopeptidase N.^44^ The hydrolysis is thought to proceed via general base-catalyzed mechanism as shown in Figure 2.^33^

**Figure 2:**
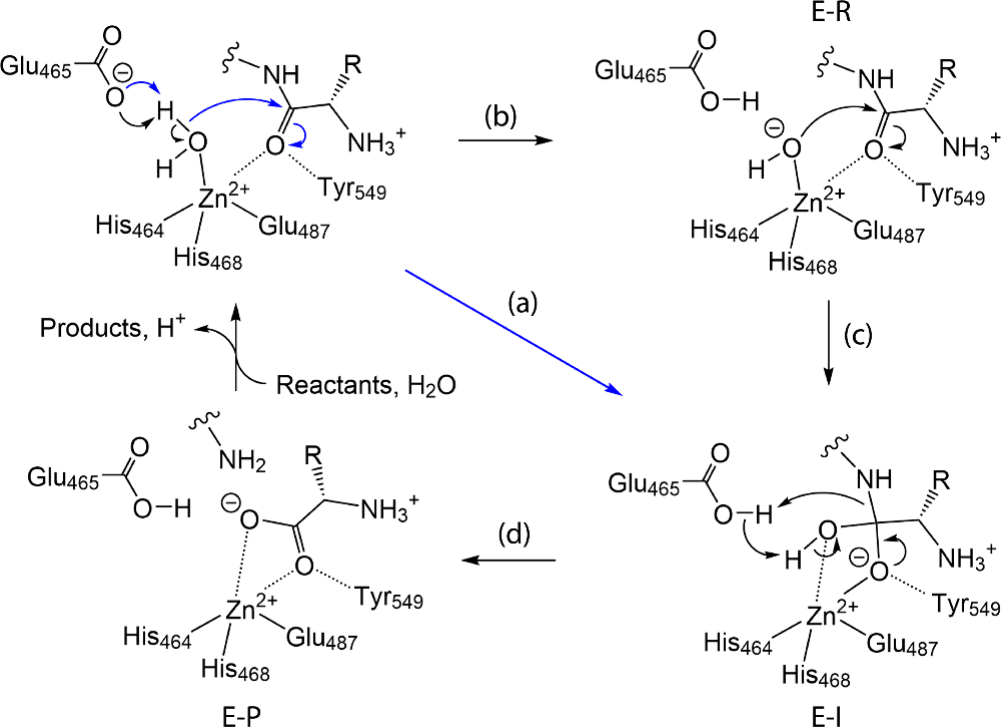
General base-catalyzed mechanism of peptide hydrolysis by IRAP (R = Cys and Val in oxytocin and angiotensin IV, respectively). (a) The mechanism includes the formation of the oxyanion tetrahedral intermediate via concerted proton transfer from a water molecule to Glu465 and the nucleophilic attack of water oxygen on the carbonyl carbon of the scissile bond (blue arrows). (b) The alternative stepwise mechanism (black arrows) includes the initial proton abstraction and (c) the subsequent nucleophilic attack of the newly formed hydroxide ion on the peptide carbonyl group generating the tetrahedral intermediate. (d) The final step is the collapse of the tetrahedral intermediate upon the proton shuttle from Glu465 to a nitrogen of the scissile bond leaving a cleaved peptide bond.

The differential catalytic activity of IRAP toward oxytocin and angiotensin IV presents a significant challenge in drug design targeting this enzyme. While oxytocin serves as a substrate for IRAP, angiotensin IV acts as an inhibitor, despite their similar binding poses in the active site. This distinction is helpful for developing effective IRAP-targeted therapies, particularly for neurological disorders.^45^ Recent advancements have led to the discovery of novel IRAP inhibitors^30,46–54^ and improved our understanding of binding affinities and structural dynamics.^55–59^ However, the molecular basis for IRAP’s contrasting behavior toward these two peptides remains unclear.

To address this knowledge gap, this study employs multiscale simulation methods, including QM/MM free energy molecular dynamics simulations (MD) and electronic structure analysis, to compare the substrate (oxytocin) and the inhibitor (angiotensin IV). Recognizing the importance of noncovalent interactions in drug discovery, our approach involves the application of QM methods to overcome limitations of the empirical force field for description of these interactions. By elucidating the molecular determinants that differentiate the inhibitor from the substrate, this research aims to provide crucial insights for rational drug design. Understanding these subtle differences could guide the development of more potent and selective IRAP inhibitors, potentially leading to improved treatments for cognitive disorders and other IRAP-related conditions.

## Methods

### Enzyme-Reactant Complex

The crystal structure of IRAP monomer in the catalytically active closed conformation with the phosphinic acid ligand was obtained from the Protein Data Bank (PDB ID 5MJ6).^60^ The missing loop 640–648 was taken from an AlphaFold model^61,62^ and a disulfide bridge was created between cysteines 828 and 835. All crystal water molecules were retained, while the ligand, carbohydrates and bromide ions present in the PDB structure were removed. The protonation states of all titratable residues under physiological conditions were assigned with the H++ server.^63^ In the absence of a crystal structure of the complex between the enzyme with oxytocin and angiotensin IV, each peptide was docked separately near the zinc ion preserving hydrogen bonding with the GAMEN loop. Angiotensin IV was docked using the HPEPDOCK server^64^ while the cyclic oxytocin peptide was docked using GOLD.^65^ See Figure S1 in the SI for the validation of docking pose. In MD simulations, the AMBER ff14SB force field^66^ was used for protein residues, and a 12–6 Lennard-Jones potential^67^ was used to model a divalent zinc ion (which was treated QM in QM/MM simulations, see below). The obtained E-R complexes were solvated with a truncated octahedron of TIP3P water molecules^68^ with a 10 Å distance between the edge of the initial solvation cell to the edge of the solvent box, using periodic boundary condition (PBC). The systems were neutralized by adding Na^+^ counterions. Details regarding the MM molecular dynamics simulations protocols are given in the SI.

### QM/MM Molecular Dynamics Simulations

After equilibration of the systems with MM MD, unrestrained quantum-mechanics/molecular-mechanics (QM/MM) minimization was carried out for each complex. The third-order extension of the self-consistent-charge density functional tight-binding (SCC-DFTB3) method was used to describe the QM region.^69,70^ The QM region consisted of the Zn^2+^ ion, the catalytic water molecule, and the side chains of Glu465, Glu431, His464, His468, Glu487 and Tyr549. In addition, for the IRAP complex with angiotensin IV, the peptide N-terminus valine residue and the neighboring tyrosine residue backbone were included in the QM region. In the case of the complex with oxytocin, the QM region included the cysteine residue, tyrosine residue backbone and the side chain of the cysteine connected to the scissile cysteine residue by a disulfide bridge (see Figure S2 in the SI for details on the QM/MM partition). More about the reaction coordinate and QM/MM MD setup can be found in the SI. The analysis of extended QM/MM MD simulations of the E-R complex can be found in Figure S3–S5 of the SI.

### Adaptive String Method QM/MM MD Simulations

The potentials of mean force (PMF) for oxytocin and angiotensin IV hydrolysis were obtained with the adaptive string method (ASM),^71^ which provides a good, flexible description of complex reactions in enzymes.^72,73^ First, the reactant (E-R) and intermediate (E-I) structures for each peptide complex were extracted from the equilibrated QM/MM umbrella sampling windows at reaction coordinate (O_Water_-C_Peptide_ distance) values of 2.7 Å and 1.5 Å, respectively. The product complexes were generated from the E-I structures by manually attaching the Glu465-bound proton to the nitrogen atom of the scissile bond. After a short unrestrained QM/MM MD simulation, this yielded a cleaved peptide bond and the second proton from the water molecule spontaneously transferred to Glu465. Five collective variables (CVs) for the ASM simulations were chosen (after testing) to represent the distances of the bonds that break and form during the reaction, as shown in Figure S6 of the SI. Initial string optimization was carried out for 30 ps until the string had converged, after which the umbrella sampling phase was performed for up to 50 ps until the standard deviation for the individual PMF profile was under 1 kcal mol^–1^.^71^ Within the ASM protocol, the E-R and E-P states were propagated using a total of 20 replica exchange simulations (10 replicas per state) for the oxytocin and angiotensin IV systems. All ASM simulations were carried out in triplicate under an NVT ensemble at 300 K using Langevin dynamics starting from different initial conditions. Structural snapshots were collected for analysis every 10 fs. The ASM simulations were performed using the *sander.MPI* module of AmberTools22.^74^ More details about the QM/MM simulation setup, umbrella sampling, higher-level DFT/MM calculations of the potential energy surface, natural bond orbital (NBO) and noncovalent interaction (NCI) analysis^75^ are given in the SI.

## Results and Discussion

### QM/MM Molecular Dynamics Simulations of E-R Complexes

QM/MM MD simulations were conducted to elucidate the mechanistic differences in IRAP’s cleavage of the natural substrate oxytocin versus the competitive inhibitor angiotensin IV. First, unrestrained simulations were employed to probe peptide-active site interactions, focusing on catalytic residue flexibility and hydrogen bonding with the conserved GAMEN loop.

Analysis of the QM/MM MD trajectories revealed conformational change of the Glu465 side chain, particularly pronounced in the oxytocin system. This catalytic glutamate plays a dual role: deprotonating the zinc-bound water molecule in the initial reaction step and facilitating the proton transfer to the scissile peptide bond nitrogen.^33,35,76^ Two distinct Glu465 rotamers were observed, designated as OE1 and OE2 conformations based on which carboxylate oxygen acts as a hydrogen bond acceptor to the catalytic water molecule (see Figure 3). These conformations were characterized by dihedral angles of approximately – 10° (eclipsed, OE1) and – 60° (staggered, OE2) formed by the side chain carbons (Figure 3a). In both oxytocin and angiotensin IV complexes, Glu465 predominantly adopted the staggered (OE2) conformation. Notably, reversible proton transfer from the catalytic water molecule to Glu465 was observed during simulations, generating a zinc-bound hydroxide, especially in the OE1 conformation. These findings suggest that subtle conformational changes of the catalytic glutamate might play a critical role in the peptide hydrolysis mechanism. Representative structures of the peptides in the active site with the Glu465 in the OE1 and OE2 conformations are depicted in Figures 3b for oxytocin and angiotensin IV.

**Figure 3:**
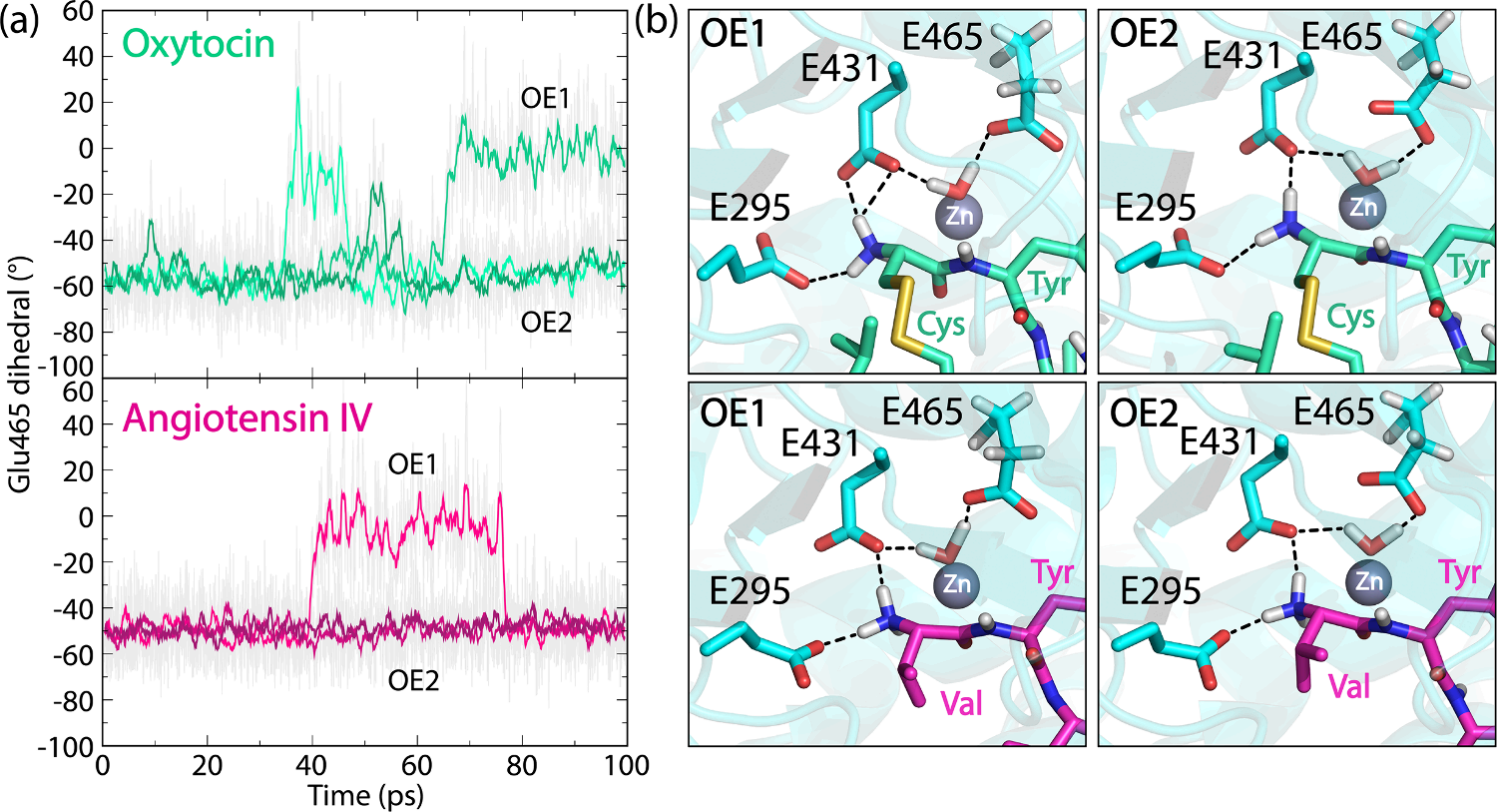
(a) Time evolution of the Glu465 side chain dihedral comprised of the Cα-Cβ-Cγ-Cδ carbon atoms calculated from three repeat unrestrained QM/MM MD simulations of the E-R complex between IRAP with oxytocin and angiotensin IV. The values of around −10 and −60° correspond to the eclipsed OE1 and staggered OE2 conformation, respectively. (b) Representative structures from QM/MM MD simulations of each complex showing the two conformations of Glu465 and hydrogen bonding network with Glu295 and Glu431. Side chain hydrogen atoms are shown to demonstrate the features of the eclipsed (OE1) and staggered (OE2) conformations of Glu465, while the backbone and the rest of the nonpolar hydrogens, as well as the zinc coordination site and the GAMEN loop are omitted for clarity.

The water molecule (or hydroxide) remained coordinated to the zinc throughout the simulation, exhibiting an average bond length of approximately 2 Å in both systems. A significantly elongated bond with a broader distribution of interatomic distances was observed between the metal and the carbonyl oxygen of the scissile peptide bond. The mean distance was marginally greater for oxytocin (3.12 Å) and displayed a larger standard deviation (0.41 Å) than for angiotensin IV (2.81 ± 0.32 Å), indicating increased conformational flexibility of oxytocin (Figure 4a and Figure S7 of the SI). These extended distances suggest that the peptides do not interact with zinc via direct coordination bonds but rather through weak attractive noncovalent interactions (NCIs) classified as a spodium bonds (SpBs).^77^ This type of interaction, wherein a Lewis base or anion donates a lone pair to the antibonding σ* orbital (sigma hole) of the metal, is prevalent in zinc-containing metalloproteins.^78^ The ubiquitous nature of SpBs in biological systems has been discussed in the literature, as evidenced by a comprehensive analysis of protein structures from the PDB that potentially contain SpBs involving Zn^2+^ ions. To elucidate the physicochemical nature of these interactions, calculations of the electrostatic potential (ESP) of the zinc sites were performed, identifying regions of positive ESP along the metal’s coordinating bonds. Interestingly, we found a remarkable similarity in the interaction between the oxygen atom of the inhibitor and the Zn^2+^ ion in metallo-β-lactamase VIM-2 ^78,79^ and between the catalytic zinc and substrate (oxytocin) or inhibitor (angiotensin IV) in IRAP (Figure S8, Supporting Information). The identification and characterization of SpBs in these enzymatic systems provide crucial insights into the mechanisms of enzyme catalysis and inhibition.

**Figure 4:**
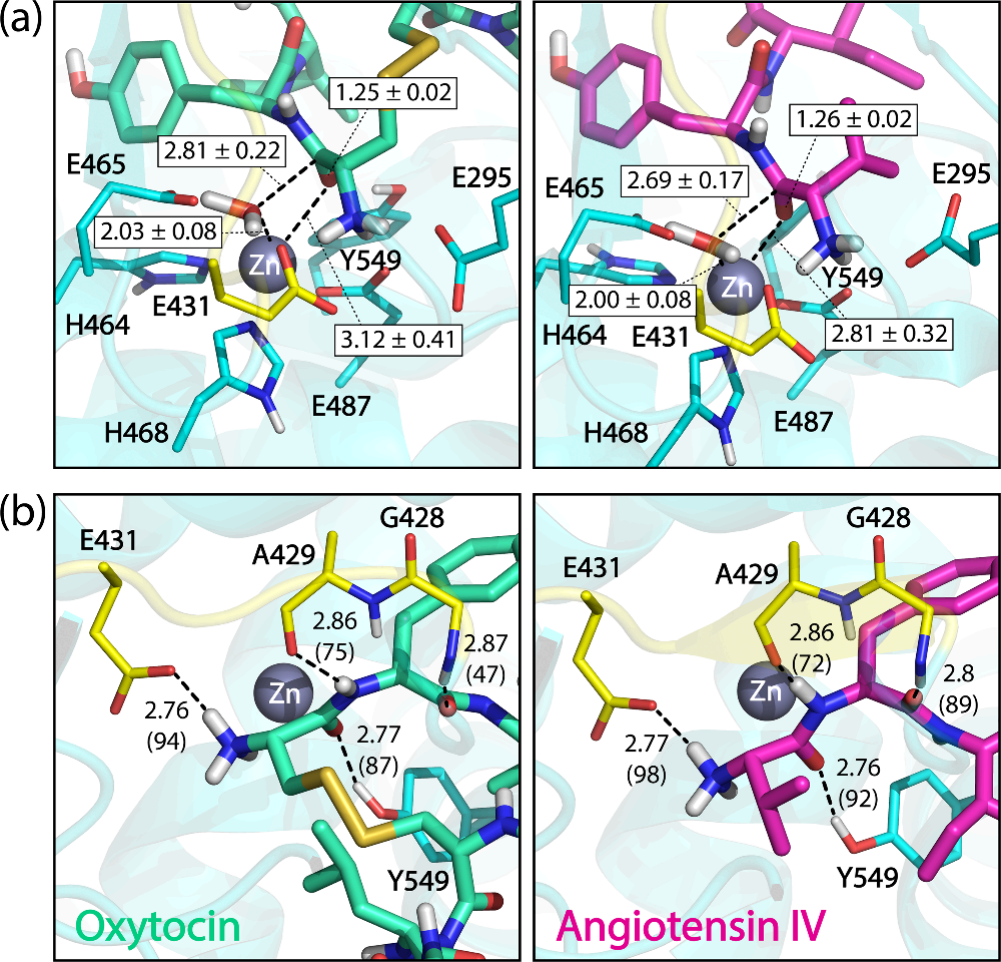
Representative structures from unrestrained QM/MM MD simulations of the E-R (reactant) complex between IRAP (OE1 conformation) with oxytocin and angiotensin IV. (a) Average distances (in Å) between the catalytic water molecule and each peptide with the zinc ion in the oxytocin and angiotensin IV systems. (b) Average hydrogen bond distances between the peptides and the GAMEN loop and the catalytic Tyr549 with percentage occupancy shown in parentheses. All distances are shown in angstrom. Only interactions with more than 10% fraction are shown.

The SpB identified in IRAP was visualized as a green isosurface between the zinc ion and oxygen of the scissile peptide bond. This interaction was characterized using NCI analysis based on the peaks observed in the reduced density gradient (RDG).^75,80^ The analysis was performed using single-point calculations at the B3LYP-D3BJ/6–31G­(d)/MM level of theory on representative structures extracted from DFTB3/MM molecular dynamics simulations (Figure S7 and Figure S9 of the SI).

Oxytocin exhibited a distinct binding pose due to its cyclic core structure, interacting with residues distal to the active site (see Figure 1b and Figure 4). Both peptides, however, demonstrated analogous N-terminal interactions, including robust hydrogen bonding between the NH_3_^+^ group and the glutamate residues Glu295, Glu431, and Glu487.^81–83^ Additionally, the scissile bond oxygen interacted with Zn^2+^ and Tyr549. The GAMEN loop facilitated multiple hydrogen bonds, notably between the scissile NH group and the Ala429 backbone carbonyl, enabling the peptides to adopt an antiparallel β-strand conformation (Figure 4b). This interaction is crucial for both inhibitor complex formation and substrate catalytic activity.^84^ It has been shown that the GAMEN loop, part of the D2 domain, undergoes substantial conformational changes upon inhibitor binding, inducing the catalytically active closed conformation of the IRAP active site.^60^ This conformational plasticity is essential for inhibitor and substrate binding.^60^ Site-directed mutagenesis studies on M1 aminopeptidases have demonstrated that mutations of Glu431 ^76^ and Tyr549 ^60^ significantly reduce the catalytic activity of IRAP and ERAP1, respectively.

Hydrogen bond and close contact analyses revealed that angiotensin IV forms more stable complex with IRAP compared to oxytocin, particularly with Gly428, exhibiting a 42% higher H-bond frequency (Figure 4b). As a competitive inhibitor, angiotensin IV likely forms stronger interactions with the active site of IRAP, resulting in a thermodynamically more stable enzyme–inhibitor complex. Further investigation was conducted through the analysis of extended QM/MM MD simulations. These simulations revealed that while both peptides exhibited high stability and remained bound in the active site, the complex with angiotensin IV demonstrated superior stability. This enhanced stability was primarily attributed to additional stacking interactions observed between the N-terminal valine residue and Phe544, as well as between the isoleucine at position 3 with both Tyr549 and Tyr961 (Figures S3–S5 in the SI). These intermolecular interactions contribute to the overall thermodynamic stability of the angiotensin IV-enzyme complex, potentially explaining its increased binding affinity and biological activity. To validate these hypotheses and elucidate the influence of Glu465 conformation, QM/MM umbrella sampling and adaptive string method (ASM) free energy simulations were conducted on both oxytocin and angiotensin IV complexes to model the hydrolysis reaction.

### QM/MM Umbrella Sampling Simulations of the Water Nucleophilic Attack

The free energy profiles exhibit significant disparities between the two peptides. A stable intermediate was observed at a reaction coordinate (O_Water_-C_Peptide_) value of 1.5 Å during the reaction with oxytocin, but not with angiotensin IV (Figure S10a and Figure S11a of the SI for the reactions with Glu465 in the OE1 and OE2 conformations, respectively). The simulations demonstrated that the reaction can proceed with the catalytic Glu465 in either the OE1 or OE2 conformation, with a slightly lower energy barrier for the OE1 conformation. These results indicate that oxytocin cleavage occurs via a concerted, asynchronous mechanism involving proton transfer from the water molecule to Glu465 and nucleophilic attack by hydroxide. The free energy barrier was calculated to be approximately 6.7 kcal mol^–1^ at a reaction coordinate value of ∼ 1.8 Å, suggesting a late transition state (Figure S10b, Supporting Information).

The tetrahedral intermediate (TI) formed in the reaction with both peptides is predominantly stabilized by the oxyanion hole, comprised of the hydrogen bond-donating hydroxyl group of Tyr549. The scissile peptide bond oxygen atom, which develops a partial negative charge during hydroxide attack, is further stabilized by the positively charged Zn^2+^ ion. Notably, the intermediate along the oxytocin reaction pathway starting from Glu465 in the OE2 conformation demonstrated the potential to adopt an alternative hydrogen bonding network, which had minimal effects on the calculated potential of mean force (PMF) (see Figure S11 of the SI). Conversely, simulations of the angiotensin IV reaction revealed that proton transfer from the zinc-bound water to Glu465 in either the OE1 and OE2 conformation yielded a higher energy intermediate, with no distinct free energy well corresponding to the tetrahedral intermediate (Figure S10 and Figure S11 in the SI).

To elucidate the underlying causes of the differences in the PMFs between oxytocin and angiotensin IV, the geometries of the tetrahedral intermediates were analyzed. Visual inspection of snapshots extracted from the ensemble of umbrella sampling windows at a reaction coordinate of 1.5 Å revealed common structural features of the tetrahedral intermediates in both peptides: the scissile NH group forms a hydrogen bond with the Ala429 backbone due to the hybridization of the scissile nitrogen, with the lone pair oriented away from the protonated Glu465. This geometry of the scissile nitrogen was also observed in the oxytocin tetrahedral intermediate, indicating that the lone pair of the scissile nitrogen must adopt an orientation away from the protonated Glu465 following nucleophilic attack to form a stable intermediate (Figure 5). The stereochemistry of the TIs in both peptides aligns with Deslongchamps’ stereoelectronic theory.^85,86^ This theory posits that a favorable orbital interaction occurs between the nitrogen lone pair and the sigma antibonding orbital of the newly formed C–O bond subsequent to the nucleophilic attack.

**Figure 5:**
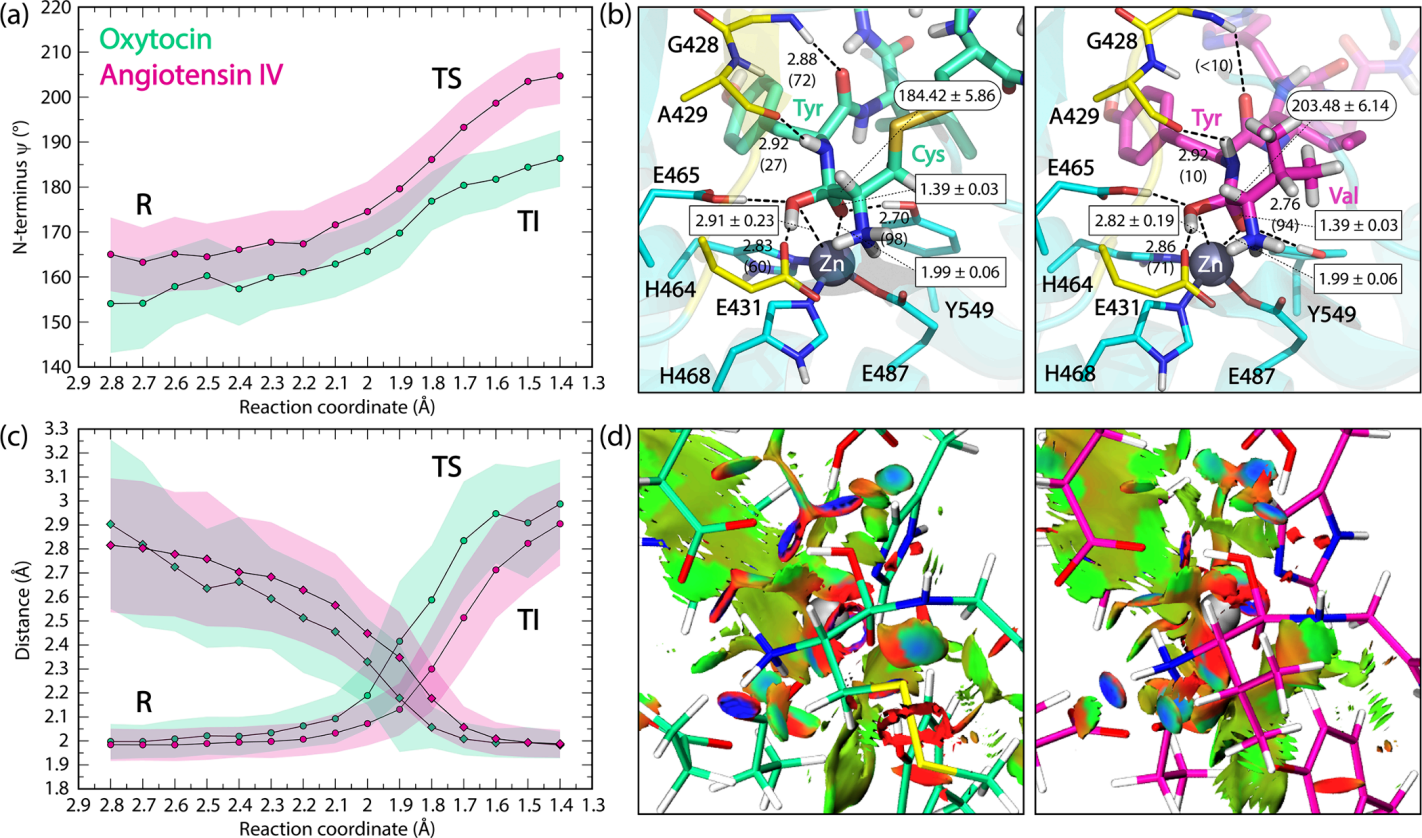
(a) ψ (N–Cα–C-N) dihedral of the peptide N-terminal residue along the reaction coordinate. Average values are shown as circles with the error (1 SD) highlighted in a transparent shaded region and are calculated for each window from three repeat umbrella simulations for both oxytocin and angiotensin IV. (b) Representative structures from umbrella windows at 1.5 Å depicting features of the tetrahedral intermediate for each peptide. Only interactions with more than 10% occupancy are shown. (c) Progression of the zinc coordination by calculating the distance between the zinc ion and the catalytic water oxygen (circle) and peptide carbonyl oxygen (diamond). (d) Noncovalent interaction (NCI) isosurfaces of the TI state indicating types of interaction. The isosurfaces are colored with a blue-green-red scale according to the values of sign­(λ_2_)­ρ, ranging from −0.035 to 0.02 au Blue indicates strong attractive (hydrogen bonds and coordinating bonds) and red indicates strong repulsive (steric effects) interaction. Green indicates weak attractive van der Waals forces (London dispersion and spodium bonds). The isovalue is set to 0.7.

The comparative analysis of oxytocin and angiotensin IV tetrahedral intermediates reveals both similarities and significant differences in their interactions with the enzyme’s active site. While both peptides form analogous hydrogen bonds with the backbones of Gly428 and Ala429, as well as the side chain of Glu431 from the GAMEN loop, notable distinctions were observed in the frequency and nature of these interactions. A quantitative analysis revealed a 17% reduction in hydrogen bonding frequency between the NH of the scissile peptide and Ala429 in the angiotensin IV complex. Additionally, a markedly lower occurrence of hydrogen bonding with Gly428 was observed in the angiotensin IV complex compared to oxytocin (Figure 5b and Figure S12 in the SI).

A critical difference between the TI states of the two peptides involves their interaction with the catalytic zinc: monodentate coordination was found between oxytocin and the Zn^2+^ ion, whereas angiotensin IV formed a bidentate coordination to the metal through both hemiketal oxygens (see Figure S13 in the SI for the evolution of key distances along the reaction coordinate). This distinction was identified through distance analysis and corroborated by noncovalent interaction (NCI) analysis of DFTB3/MM umbrella sampling snapshots shown in Figure 5d. As a result of different coordination to Zn^2+^, the N-terminal ψ dihedral of each peptide adopts different conformations (Figure 5 and Figure S13 in the SI), which leads to steric clash between the bulkier valine side chain with the lone pair of hybridizing scissile nitrogen, potentially contributing to destabilization of the angiotensin IV tetrahedral intermediate.^87^ These structural differences suggest that the subsequent proton transfer from Glu465 to the scissile nitrogen, which requires improved orbital overlap, would necessitate not only the inversion of the scissile nitrogen but also breaking of the strong bidentate coordination to the zinc ion in TI of angiotensin IV. This process likely imposes a high energy barrier, potentially impeding the reaction’s progression from this intermediate state. Similar bidentate zinc coordination was found in the case of nonhydrolyzable transition state analogs as inhibitors of ERAP1 ^11^ and metallo-β-lactamases.^88^ The importance of N-terminal residues in binding affinity is underscored by experimental data. Mutation of the N-terminal valine in angiotensin IV to cysteine resulted in reduced *K*_i_, suggesting that the terminal valine residue may be crucial for the high affinity of angiotensin IV for IRAP. This observation aligns with the structural features of the majority of known angiotensin IV-based inhibitors.^30,52,89^

### QM/MM Adaptive String Method Simulations of Peptide Cleavage

The comparative analysis of umbrella sampling and adaptive string method QM/MM simulations for calculating free energy profiles associated with tetrahedral intermediate formation reveals distinct advantages of the ASM approach. While both methods were employed, only ASM facilitated the exploration of the reaction along the true minimum free energy path, leading to complete hydrolysis of oxytocin and angiotensin IV peptides. This superiority is attributed to ASM’s ability to incorporate multiple collective variables simultaneously.

The ASM simulations show a marked difference in reactivity between the two peptides with IRAP. Specifically, the activation free energies for hydrolysis were determined to be 21.5 kcal mol^–1^ and 27.6 kcal mol^–1^ for oxytocin and angiotensin IV, respectively (Figure 6). Qualitatively consistent PMF profiles were observed in ASM simulations initiated from the catalytic Glu465 in OE2 conformation (see Figure S14, Supporting Information).

**Figure 6:**
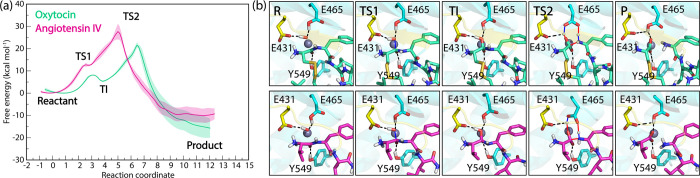
(a) Potential of mean force (PMF) for N-terminal peptide bond cleavage in oxytocin (green) and angiotensin IV (magenta) obtained from ASM QM/MM MD simulations starting from the OE1 conformation of Glu465. The average free energy profiles (thick lines) were calculated from three repeat profiles with the standard deviation shown as transparent shaded regions. (b) Representative structures of the intermediates and transition states along the hydrolysis coordinate. The zinc ion is shown as gray sphere and the GAMEN loop is shown in yellow cartoon representation. Glu431, Glu465, and Tyr549 side chains are shown. Bonds forming and breaking in the transition state are shown as blue and red lines, respectively. The reaction coordinate is given in a.m.u.^1/2^Å.

The ASM-derived free energy barriers for the second reaction step, involving concerted protonation of the scissile nitrogen and C–N bond cleavage, appear to be comparable for both peptides. This suggests that the key determinant of their differential reactivity in IRAP resides in the stability of the tetrahedral intermediate formed during the initial reaction step. Interestingly, the identification of a significant disparity between the natural substrate and inhibitor in the TS1 and TI states, which is not apparent in the higher energy barrier state TS2 (conventionally targeted in the transition state analogue drug design),^33^ presents a novel avenue for IRAP inhibitor design. By targeting interactions unique to the TS1 of angiotensin IV, as elucidated by our QM/MM simulations, a more selective yet broadly applicable template for inhibitor design may be developed.

### Higher-Level DFT/MM Calculations and Electronic Structure Analysis

Consistent with the DFTB3/MM enhanced sampling simulations of the nucleophilic attack reaction, similar geometries and reaction energetics were obtained using a static DFT/MM approach. The QM region was treated at the B3LYP-D3BJ/def2-TZVP level of theory. Geometry optimizations were performed with Py-ChemShell package^90^ at the B3LYP-D3BJ/6–31G­(d):MM level (see SI for details). The QM/MM calculations yielded an energy barrier of 10.6 kcal mol^–1^ for the formation of a stable TI in the case of oxytocin, with the TI lying 6.1 kcal mol^–1^ above the reactant state. For angiotensin IV, the formation of the TI was less favorable, with an energy barrier of 16.0 kcal mol^–1^ and a reaction energy of 15.2 kcal mol^–1^ (see potential energy profiles in Figure S15, Supporting Information).

Natural bond orbital (NBO) analysis (Figure S16, Supporting Information) provided insights into the electronic structure differences between the two peptides. For oxytocin, overlap between the lone pair of the peptide nitrogen atom and the antibonding σ orbital of the newly formed C–O bond stabilizes the TI by approximately 11.2 kcal mol^–1^. In angiotensin IV, this stabilization is more pronounced at 18.9 kcal mol^–1^. The NBO analysis also revealed a unique stabilizing interaction (∼7 kcal mol^–1^) in oxytocin between the hybridizing nitrogen lone pair and the antibonding σ* of the disulfide Sγ-Sγ’ bond. This interaction was observed exclusively in the TI state of oxytocin. Both the DFT/MM optimized geometries and DFTB3/MM umbrella sampling snapshots showed a close contact between the scissile nitrogen and the sulfur atom of the disulfide bond in the oxytocin tetrahedral intermediate. This interaction was visualized using NCI isosurfaces between Npep and Sγ (Figure 5d and Figure S16, Supporting Information). A similar type of interaction has previously been found in other proteins where it was proposed that the engagement of the S–S bonds in a close contact with a carbonyl O atom increases the susceptibility for their reduction.^91^

These findings suggest that the mechanism of hydrolysis in IRAP likely involves nitrogen inversion,^92,93^ which is stabilized by a hydrogen bond with Ala429 from the GAMEN loop. The unique S···N interaction observed in oxytocin may contribute to its enhanced reactivity compared to angiotensin IV. The enhanced stability of the TS1 and TI with the substrate can be attributed to a favorable interaction between the sp^2^-hybridized nitrogen and the sulfur atom of the disulfide bond. However, elevated overall barriers for inhibitor cleavage are elucidated by multiple factors: stabilization of the angiotensin IV E-R state via hydrogen bonding with the GAMEN loop, favorable stacking between N-terminal valine and isoleucine at the position 3 with the neighboring aromatic residues, destabilization of the E-I complex due to steric clash between the bulky valine side chain and the hybridizing nitrogen and significant peptide strain, manifested through deviation of the valine C–Cα-Cβ angle and the N-terminus ψ dihedral from the ideal conformation (see Figure S16 and Table S1 in the SI).

The potential energy profiles computed at the B3LYP/MM level of theory for the nucleophilic attack and peptide bond cleavage steps indicate that the DFTB3 method provides a reasonable description of both geometries and relative energetics for the IRAP-catalyzed reactions with oxytocin and angiotensin IV (Figure S17, Supporting Information). DFTB3 captures the essential features of the reaction mechanism, including key transition state and intermediate structures, in a manner that is broadly consistent with higher-level (B3LYP/MM) calculations. This general agreement supports the validity of our computational approach and lends confidence to the conclusions. Notably, the activation barriers calculated for oxytocin and angiotensin IV cleavage at the B3LYP/MM level were 20.5 and 36.9 kcal mol^–1^, respectively, values that are similar to those obtained with DFTB3/MM (21.5 and 27.6 kcal mol^–1^ for oxytocin and angiotensin IV, respectively). This level of agreement suggests that DFTB3 is a suitable and computationally efficient alternative to more demanding DFT methods for modeling these reactions. The reaction energies do apparently differ, being more exothermic/exergonic from DFTB3/MM dynamics, but marginally endothermic from B3LYP/MM potential energies: this is probably due to the limited sampling and structural relaxation in the static calculations.

The catalytic efficiency of IRAP in oxytocin cleavage has been experimentally determined by Mpakali et al.^59^ who reported a *k*_cat_ of approximately 0.45 s^–1^, which translates to an activation barrier of ∼ 18 kcal mol^–1^ at 300 K. Our QM/MM methods yielded activation barriers of 21.5 kcal mol^–1^ using DFTB3 and 20.5 kcal mol^–1^ using B3LYP-D3/def2-TZVP, demonstrating good agreement with the experimental data within the error of the method. See Table S2 and Figure S18 for summarized energies computed with different approaches.

## Conclusions

The inhibition of insulin-regulated aminopeptidase (IRAP) has been demonstrated to increase levels of neuroactive peptides in the brain, thereby enhancing long-term potentiation and improving memory retention. In this study, enhanced sampling QM/MM MD simulations were employed to elucidate the free energy landscape underlying the differential hydrolysis of the natural substrate oxytocin and the prototypical peptide inhibitor angiotensin IV by IRAP.

The conformational flexibility of the catalytic Glu465 and peptide interactions with the GAMEN loop were identified as critical determinants of peptide reactivity. The reaction mechanism involves a two-step proton transfer process, initiated by the Zn^2+^-bound water molecule. The OE1 conformation of Glu465 was found to facilitate proton transfer to the scissile nitrogen in the second reaction step. Unrestrained simulations revealed superior hydrogen bonding of angiotensin IV within the IRAP active site, particularly with Gly428 and Ala429 of the GAMEN loop. This tighter complex formation contributes to an increased activation energy barrier for angiotensin IV hydrolysis.

Adaptive string method QM/MM simulations of the complete peptide hydrolysis along the minimum free energy path revealed a significant difference in activation free energy of approximately 6.1 kcal mol^–1^ between oxytocin and angiotensin IV. Oxytocin cleavage was found to proceed via a concerted but asynchronous mechanism, forming a tetrahedral intermediate stabilized by electron donation from the scissile nitrogen lone pair to the σ-hole of the adjacent disulfide bond. In contrast, angiotensin IV did not form a stable intermediate, exhibiting distinct geometric features in its tetrahedral intermediate. These included bidentate zinc coordination and unfavorable conformational changes, resulting in steric repulsion between bulky valine side chain and hybridizing nitrogen atom’s lone pair. These distinctive intermolecular interactions were elucidated through the application of higher-level electronic structure calculations in the enzyme environment. The characterization was primarily conducted utilizing natural bond orbital (NBO) theory and noncovalent interaction (NCI) analysis.

Our investigation elucidates the significance of noncovalent spodium bonds (SpBs) as an emerging paradigm in rational drug design, offering novel strategies for the optimization of pharmaceutical compounds. These interactions exhibit potential for modulating protein–ligand binding affinities and can be exploited to fine-tune molecular recognition processes. The integration of SpBs into computational models, particularly in QM/MM simulations, enhances the predictive power of in silico drug discovery pipelines. QM/MM methods allow for the examination of SpBs along reaction coordinates and intermediates, providing crucial insights into their role in enzymatic mechanisms and inhibitor binding. This approach has demonstrated efficacy in elucidating the binding modes of peptides such as oxytocin and angiotensin IV to IRAP, thereby advancing our understanding of structure–activity relationships in drug-target interactions. The incorporation of SpBs into molecular design strategies represents a promising avenue for the development of more potent and selective therapeutic agents.

The findings of this study provide valuable insights into the molecular basis of IRAP substrate specificity and inhibitor binding. The presence of a bulky, hydrophobic N-terminal residue, such as valine, may contribute to inhibitor binding specificity through hydrogen bonding with the GAMEN loop^94^ while impeding peptide hydrolysis by modulating tetrahedral intermediate geometry and enzyme interactions. These mechanistic insights have significant implications for the rational design and development of potent IRAP inhibitors as potential therapeutic agents.

## Supplementary Material



## Data Availability

The representative QM/MM molecular structures and AMBER topologies, coordinates and input files are available on Zenodo https://doi.org/10.5281/zenodo.15236907.

## Notes

Additional simulation details, materials, and methods, including figures, plots, and tables of QM/MM simulations (PDF)

## #

Pharmaron Discovery & Early Development, West Hill Innovation Park, Hertford Road, Hoddesdon, Hertfordshire, EN11 9FH, United Kingdom (R.M.T.)
