# Supplementary material for: Quantum Mechanics/Molecular Mechanics Simulations Distinguish Insulin-Regulated Aminopeptidase Substrate (Oxytocin) and Inhibitor (Angiotensin IV) and Reveal Determinants of Activity and Inhibition
Source: J Chem Inf Model. 2025 Jun 11;65(12):6261–72. doi: 10.1021/acs.jcim.5c00869 (PMC12199304; doi:10.1021/acs.jcim.5c00869)
Supplement: Supplementary file 1 [file ci5c00869_si_001.pdf]

Supporting Information

QM/MM simulations distinguish insulin-regulated aminopeptidase substrate (oxytocin) and inhibitor (angiotensin IV) and reveal determinants of activity and inhibition

Marko Hanževački[‡], Rebecca M. Twidale[‡], Eric J. M. Lang[‡], Will Gerrard[§], David W. Wright[§], Vid Stojevic[§] and Adrian J. Mulholland[*,‡]

[‡]Centre for Computational Chemistry, School of Chemistry, University of Bristol, Bristol BS8 1TS, United Kingdom

[§]Kuano, Hauxton House, Mill Scitech Park, Mill Lane, Cambridge CB22 5HX, United Kingdom

*Corresponding author email: Adrian.Mulholland@bristol.ac.uk

Computational methods

Docking validation. To validate our docking pose we carried out two independent test. In the first test, we compared our docking pose with a solved crystal structure of the structurally related human aminopeptidase N (a member of the M1 family) in complex with angiotensin IV (PDB IDs: 4FYS and 4FYQ).[1] For the second test, we employed AlphaFold 3 (AF3)[2] to predict the structure of the multimeric complex between IRAP and angiotensin IV in the presence of Zn^{2+}. The best-ranked AF3 model exhibited high confidence metrics for the complex (ipTM = 0.83 and pTM = 0.94). The structures obtained with both approaches were aligned with out docking pose and it was confirmed that they agree well with each other (**Figure S1**).

MM molecular dynamics simulations. The prepared E-R systems were initially energy minimized using molecular mechanics (MM), with positional harmonic restraints and a force constant of 50 kcal mol^{-1} $Å^{-2}$ applied to all heavy atoms of the solute. This removed any bad contacts in the docking poses. In the initial MD simulations, the temperature of the systems was gradually increased to 310 K over 100 ps, and then was kept constant for another 100 ps of NVT simulation. The temperature was controlled using the Langevin thermostat with the collision frequency of 2 ps^{-1}. In the second step of the relaxation phase, the systems were simulated at 310 K over 100 ps at a constant pressure of 1 bar (NPT), allowing the density of the box to equilibrate using the Berendsen barostat. In both steps of the relaxation, the harmonic positional restraints with a force constant of 50 kcal mol^{-1} $Å^{-2}$ were kept only on heavy atoms of residues directly bound to the zinc ion including the metal ion to avoid any non-physical changes of the metal coordination site as a result of the MM force field description. All MM MD simulations were performed using the *pmemd.cuda* module of the AMBER18 software package.[3]

QM/MM molecular dynamics simulations. The number of QM atoms (including link hydrogens) was 114 and 115 in the oxytocin and angiotensin IV systems, respectively. The total charge and the multiplicity of the QM region in both system were −1 and 1, respectively. For the complete list of QM atoms see **Figure S2**. Each system was heated by increasing the temperature to 310 K over 15 ps. The temperature was controlled using the Langevin thermostat with a collision frequency of 5 s^{-1}. The systems were further equilibrated over 15 ps maintaining constant pressure using a Monte Carlo barostat[4] with the isotropic position scaling. Finally, three independent repeat production QM/MM MD simulations of 100 ps were carried out for each E-R complex at a constant temperature of 310 K and the constant pressure of 1 bar giving rise to a total of 300 ps per system, saving snapshots every 50 fs. Three additional ~400 ps simulations were performed to explore conformational flexibility and stability of the E-R complex with oxytocin and angiotensin IV. The analysis of these simulations is shown in **Figure S3-S5**. All QM/MM simulations were carried out using the *sander.MPI* module of the AMBER18 program.[3] Long-range electrostatic interactions were treated with the Particle Mesh Ewald (PME) approach with the non-bonded cutoff set to 12 Å. A time step of 1 fs was used in all simulations. The analysis was performed with the *cpptraj* module of the AMBER18 program.[3] Visualization was carried out using Pymol 2.5.4[5] and VMD 1.9.3.[6] Non-covalent interaction (NCI) analysis[7] based on the reduced density gradient (RDG)[8] was carried out in Multiwfn[9] using one representative geometry of E-R and E-I from DFTB3/MM simulations and performing a single-point calculations at the B3LYP-D3BJ/6-31G(d)/MM level of theory in Gaussian 16 software.[10] The cutoff distance value of 3 Å and angle value of 135° was used for the hydrogen bonds calculations. The cutoff for the close contacts calculations was 3 Å.

Umbrella sampling QM/MM MD simulations. The free energy profiles have been calculated at the DFTB3/MM level of theory due to the computational efficiency allowing extensive sampling with a comparable accuracy to DFT methods.[11] The QM/MM umbrella sampling MD simulations for the reaction of IRAP with oxytocin and angiotensin IV were performed using the same QM region as in the case of unbiased simulations. The nucleophilic attack reaction coordinate was defined as the distance between the catalytic water oxygen (O_{Water}) and the carbonyl carbon atom of the scissile peptide bond ($C_{Peptide}$). Initial structures for umbrella sampling were generated by driving the system along the predefined reaction coordinate from the E-R to the E-I state using QM/MM steered MD simulations with the force constant of 50 kcal mol^{-1} $Å^{-2}$ and a pulling speed of around 0.15 Å ps^{-1}. The distance was incremented from 2.8 Å to 1.4 Å in steps of 0.1 Å with a bond force constant of 200 kcal mol^{-1} $Å^{-2}$. The equilibrated snapshots were simulated for 10 ps per window using the NPT ensemble at 310 K and 1 bar employing the Langevin dynamics. The average potential of mean force (PMF) were calculated using the weighted histogram analysis method (WHAM)[12] on three repeat umbrella simulations. The overlap between the neighboring umbrella histograms is shown in **Figure S19** and **Figure S20** for the reaction with Glu465 in OE1 and OE2 conformation, respectively.

High-level DFT QM/MM calculations. Representative QM/MM MD snapshots of IRAP with oxytocin and angiotensin IV bound were processed in *cpptraj* to obtain non-periodic systems for QM/MM calculations. The solvation shell around the QM region was created by retaining the closest 1000 water molecules. The active region (atoms allowed to move during the geometry optimization) included all residues whose atoms were within 6 Å of QM region while the positions of all other atoms were fixed. The QM region included the Zn^{2+} ion, the catalytic water molecule, and the sidechains of Glu465, Glu431, His464, His468, Glu487, Tyr549 and the part of oxytocin and angiotensin IV similar to the case of QM/MM MD simulations. The MM atoms directly bound to the QM atoms were replaced by link hydrogen atoms generated with covalent coupling. The total charge and the multiplicity of the QM region was –1 and 1, respectively. QM/MM geometry optimization was performed with the electrostatic embedding and additive scheme at the B3LYP/MM level of theory (B3LYP/6-31G(d):AMBER) including the Grimme's D3 dispersion correction and

Becke-Johnson damping (D3BJ).[13,14] The QM/MM system was optimized to a minimum using the L-BFGS algorithm with a constant trust radius. Initial transition state search was carried out with the climbing image nudged elastic band (CI-NEB) method and reoptimized with the dimer method. Single point calculations were carried out on the previously optimized geometries using the B3LYP-D3BJ/def2-TZVP level of theory. The frequency calculations were carried out at the geometry optimization level of theory confirming that the optimized structures are transition state or true minima. All QM/MM calculations were performed using the ORCA 5.0.3/DL_POLY 5 interface in Py-ChemShell 21.0.3.[15,16] The natural bond orbital (NBO) analysis was carried out on the QM region extracted from QM/MM optimized geometries using NBO 3.1 available in Gaussian16 program.[10,17]

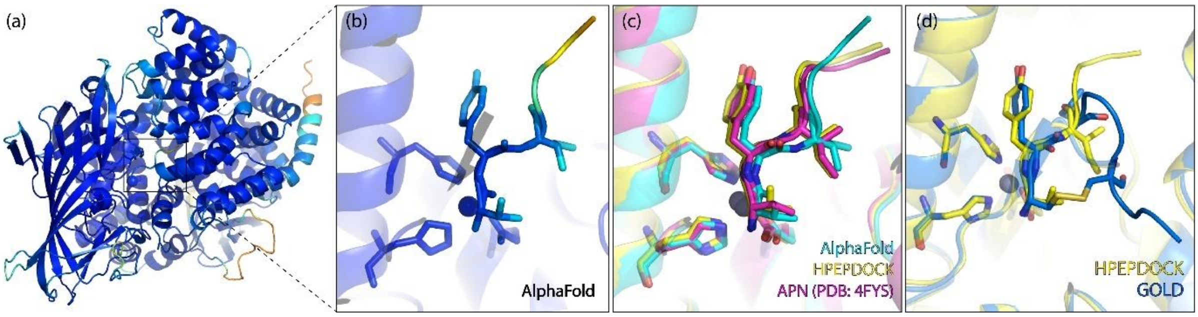

Figure S1 (a) AlphaFold 3 predicted structure of the complex between IRAP and angiotensin IV (ribbons are colored with orange-yellow-cyan-blue scheme indicating the quality of structure prediction from more confident to less confident where blue is 1 and orange is 0.6). (b) Close view of the peptide interaction with the zinc in the active site obtained from the AF3 model. (c) Superimposed structures of IRAP (obtained with AlphaFold 3 and HPEPDOCK) and X-ray structure of APN with angiotensin IV. (d) Comparison between angiotensin IV and oxytocin docking pose showing a good overlap of the N-terminus region and analogous interaction with the zinc ion.

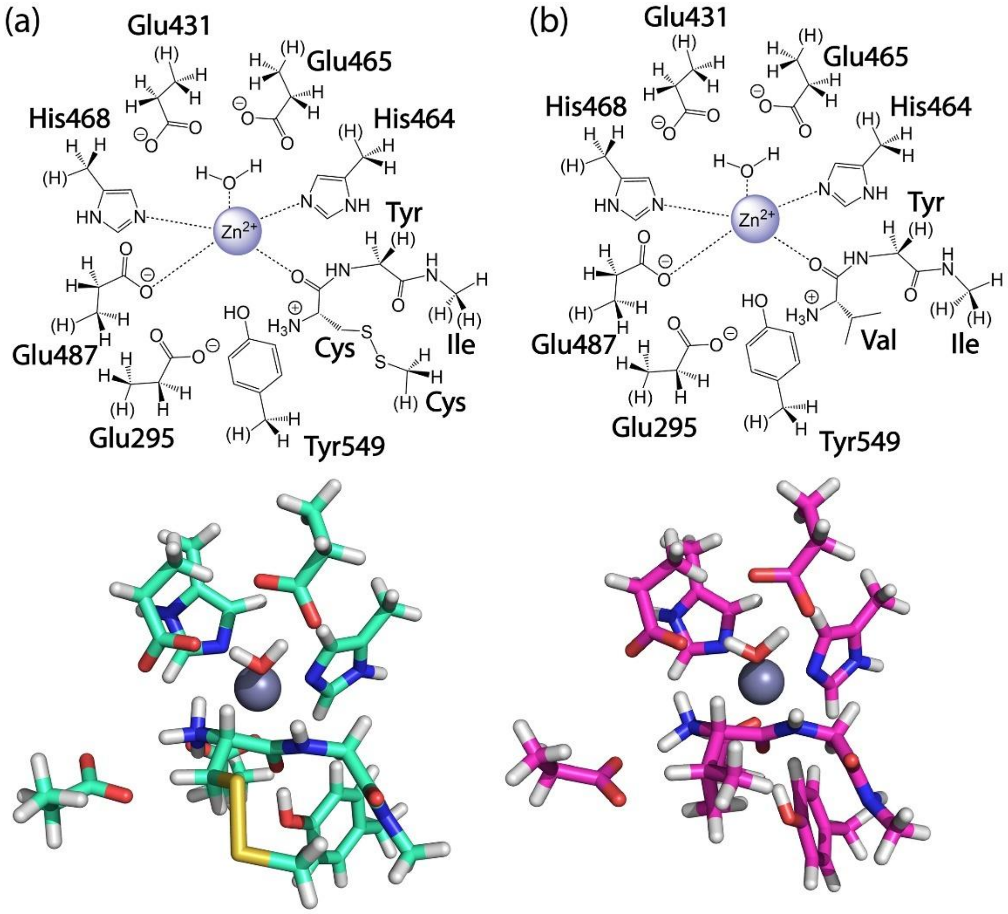

Figure S2 Atoms included in the QM region with link hydrogens shown in parentheses for (a) oxytocin and (b) angiotensin IV systems. Three dimensional structures are shown under the corresponding molecular structures.

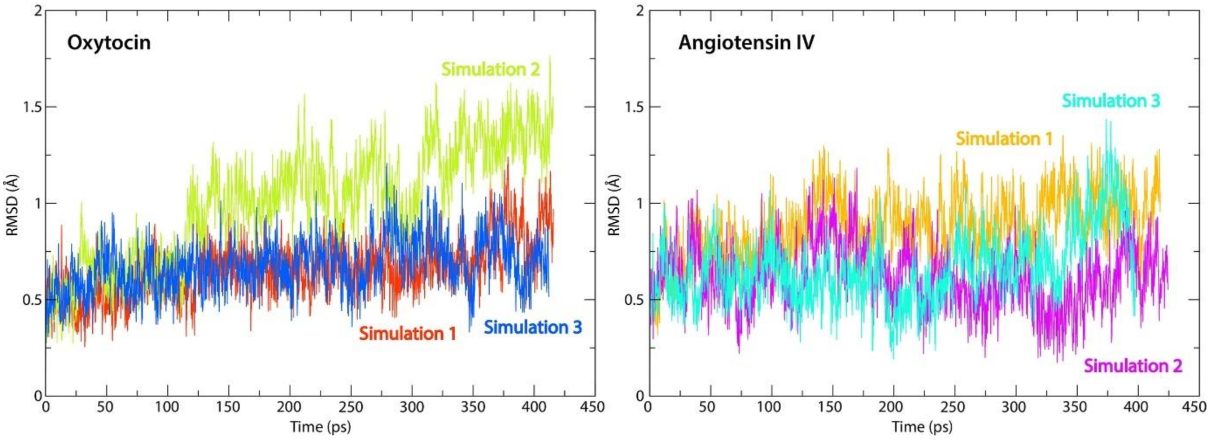

Figure S3 Root mean square deviation (RMSD) of oxytocin and angiotensin IV Cα backbone atoms extracted from extended QM/MM MD simulations and calculated from the corresponding reference structure.

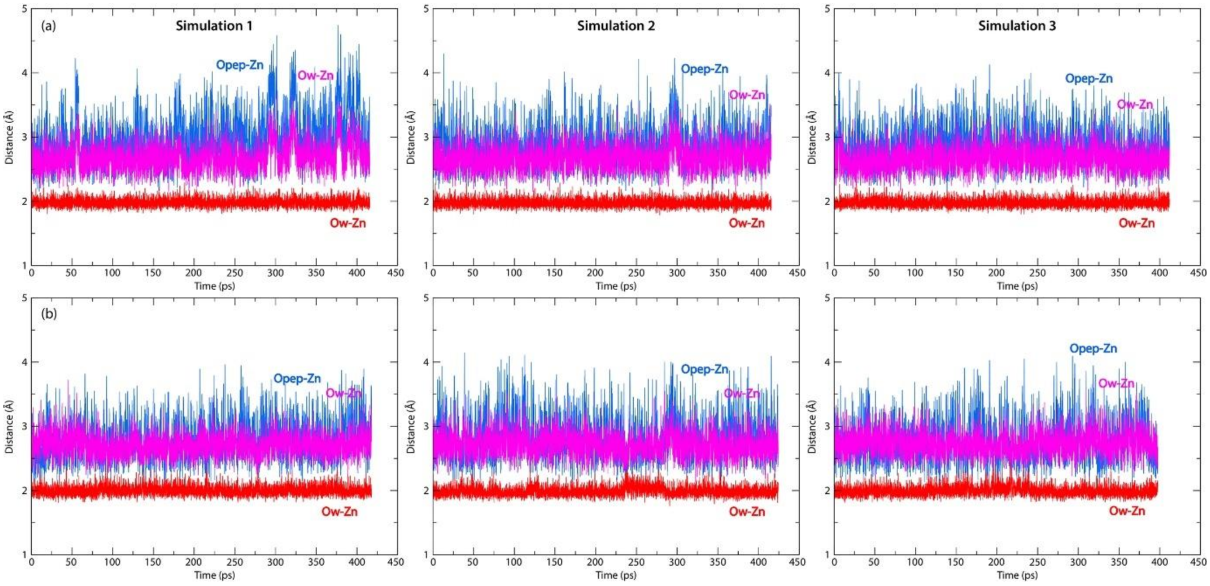

Figure S4 Key distances between (a) oxytocin and (b) angiotensin IV with Zn^{2+} and catalytic H_2O molecule in the active site of IRAP calculated from extended QM/MM MD simulations of the E-R complex.

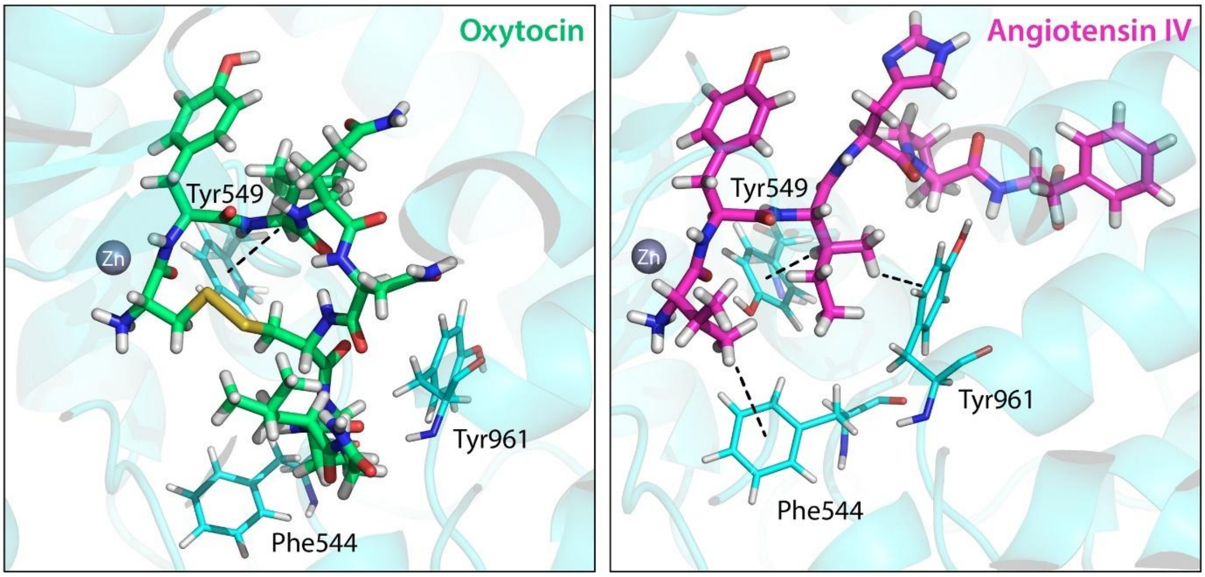

Figure S5 Representative QM/MM MD snapshots of oxytocin and angiotensin IV in the active site of IRAP.

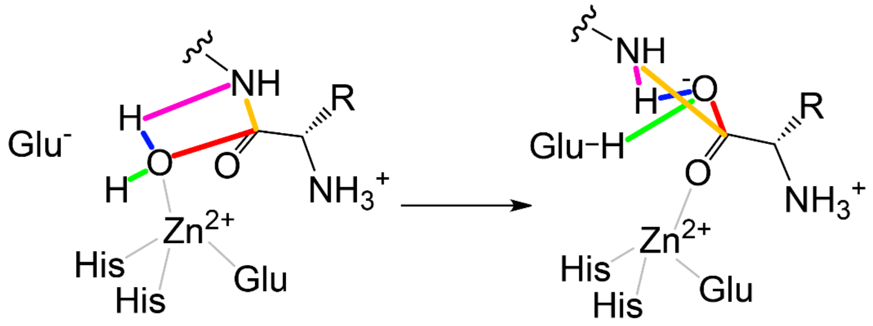

(a)	R = Cys				(b)	R = Val			
	$Glu465_{OE1}$		$Glu465_{OE2}$			$Glu465_{OE1}$		$Glu465_{OE2}$	
	E-R	E-P	E-R	E-P		E-R	E-P	E-R	E-P
O-H	1.46	2.94	1.12	3.23	O-H	1.30	3.92	1.52	2.99
O-H	0.96	1.67	0.96	1.67	O-H	0.97	2.00	0.98	3.87
C-O	2.92	1.25	3.00	1.23	C-O	2.61	1.28	2.39	1.29
N-H	3.82	1.06	3.81	0.98	N-H	3.61	1.05	3.36	1.00
C-N	1.40	3.35	1.42	3.74	C-N	1.34	3.74	1.38	2.67

Figure S6 Collective variables used in the adaptive string QM/MM MD simulations for the E-R and E-P states in (a) oxytocin and (b) angiotensin IV systems. All distances are given in angstrom.

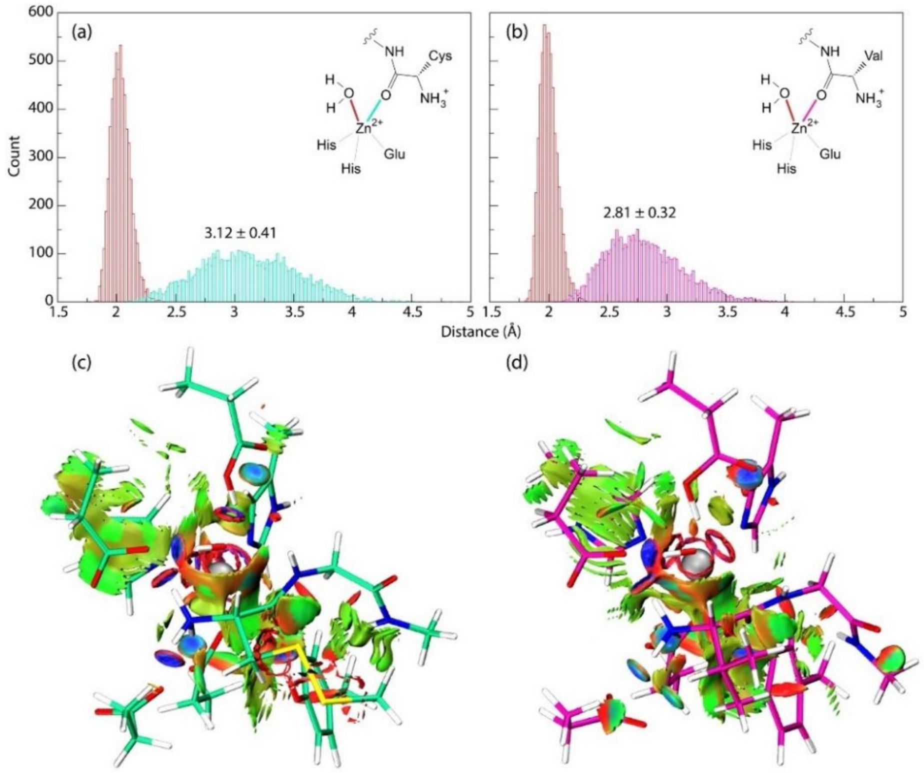

Figure S7 Histograms of distance between the catalytic zinc ion and water oxygen (red) and scissile peptide bond carbonyl oxygen from (a) oxytocin (green) and (b) angiotensin IV (magenta). Distances were calculated from unrestrained QM/MM MD simulations of the E-R complex. The non-covalent interaction (NCI) isosurfaces of the E-R complex between IRAP with (c) oxytocin and (d) angiotensin IV. The isosurfaces are depicted with a blue-green-red scale according to the values of sign(λ_2)ρ, ranging from -0.035 to 0.02 a.u. Blue indicates strong attractive (hydrogen bonds and coordinating bonds) and red indicates strong repulsive (steric effects) interaction. Green indicates weak attractive Van der Waals forces (London dispersion and spodium bonds with the metal). The isovalue of 0.7 was used.

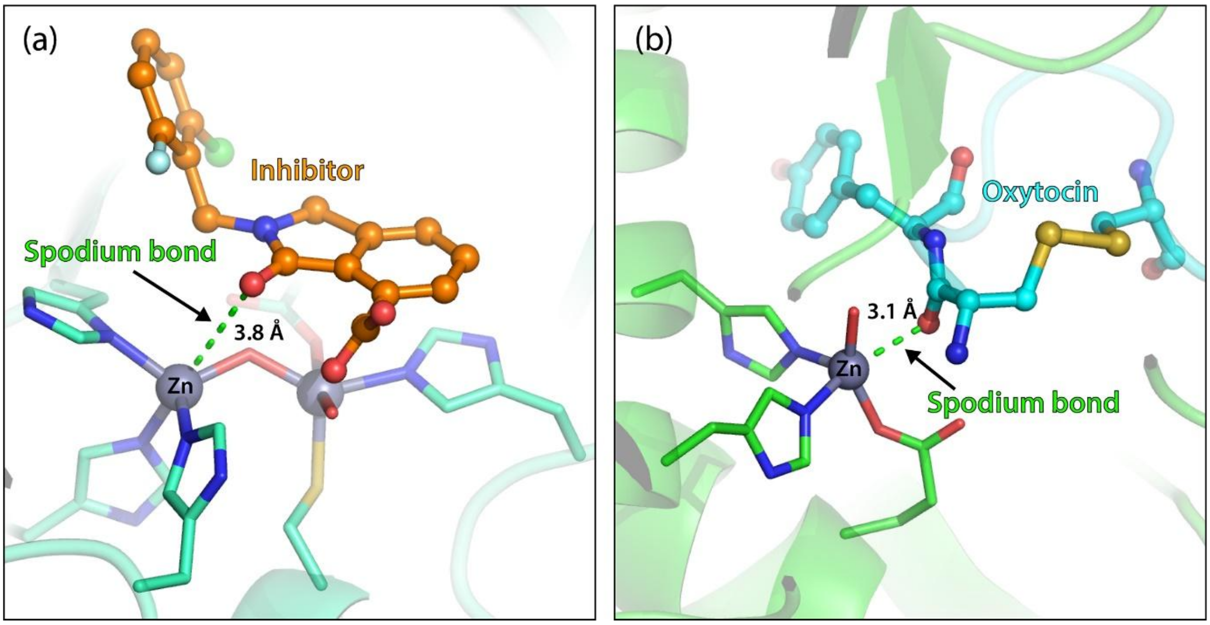

Figure S8 (a) Crystal structure of inhibitor bound to Zn^{2+} through a spodium bond in metallo-β-lactamase VIM-2 (PDB ID: 5LE1).[18] (b) The QM/MM MD snapshot depicting a spodium bond between oxytocin and Zn^{2+} in IRAP.

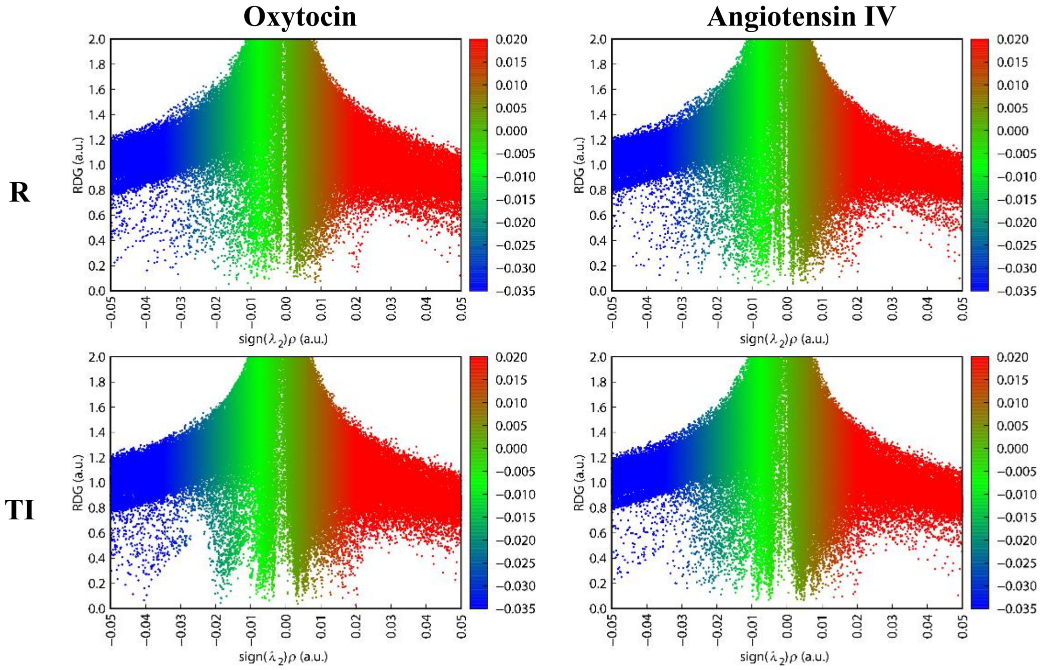

Figure S9 Reduced density gradient (RDG) scatter plots are depicted with a blue-green-red scale according to the values of sign(λ_2)ρ, ranging from -0.035 to 0.02 a.u. Blue indicates strong attractive (hydrogen bonds and coordinating bonds) and red indicates strong repulsive (steric effects) interaction. Green indicates weak attractive Van der Waals forces (London dispersion and spodium bonds with the metal).

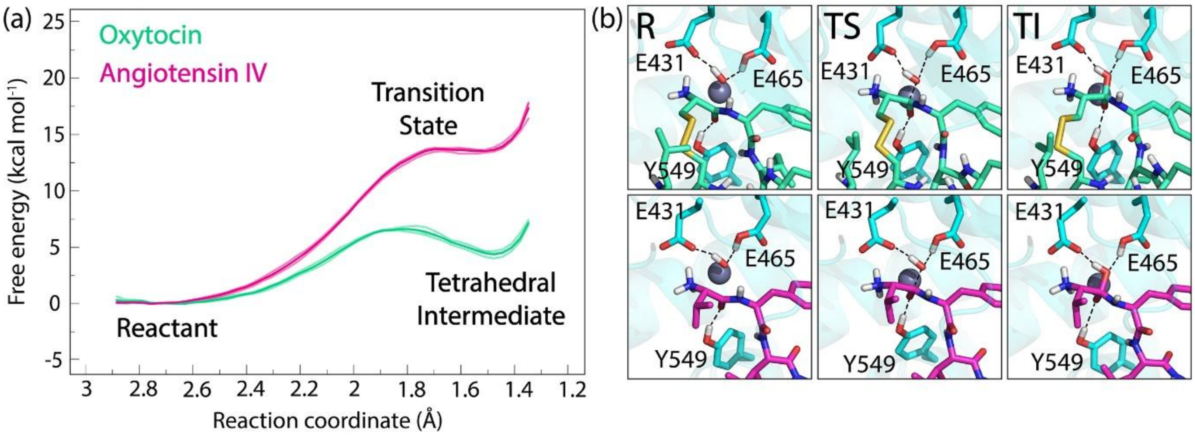

Figure S10 (a) Potential of mean force (PMF) for the Glu465-catalyzed (OE1 conformation) nucleophilic attack of oxygen from water on the carbonyl carbon of the scissile peptide bond in oxytocin (green) and angiotensin IV (magenta) obtained from umbrella sampling QM/MM MD simulations. The average free energy profiles (thick lines) were calculated from three independent repeat profiles (thin transparent lines). (b) Representative structures from umbrella windows at a particular value of the reaction coordinate. The R and TI snapshots were taken from 2.7 Å and 1.5 Å for both peptides while the TS snapshots were taken from 1.8 Å and 1.7 Å for oxytocin and angiotensin IV, respectively. The Zn^{2+} ion is shown as gray sphere. Only important side chains were shown while the rest of the active site residues and non-polar hydrogens weas omitted for clarity. All PMFs have been calculated at 310 K.

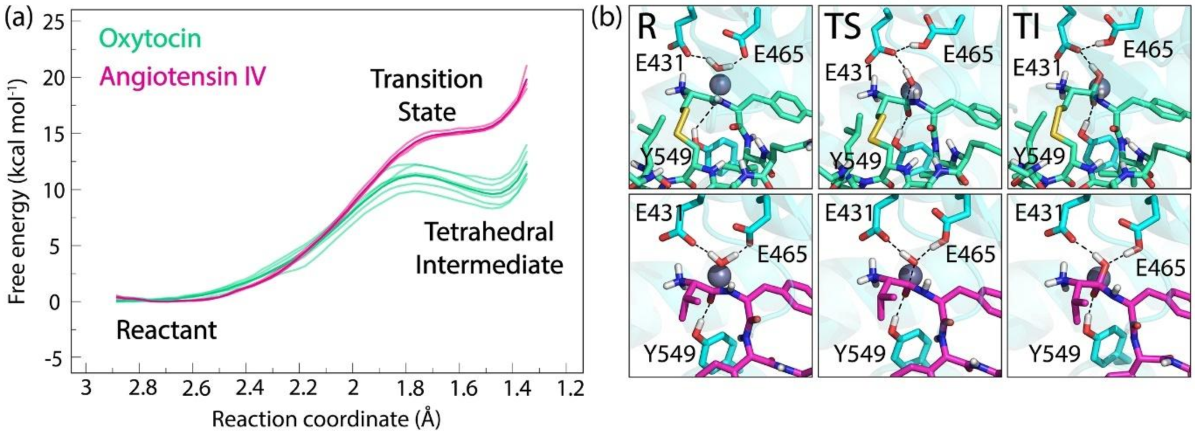

Figure S11 (a) Potential of mean force (PMF) for the Glu465-catalyzed (OE2 conformation) nucleophilic attack of oxygen from water on the carbonyl carbon of the scissile peptide bond in oxytocin (green) and angiotensin IV (magenta) obtained from umbrella sampling QM/MM MD simulations. The average free energy profiles (thick lines) were calculated from six and three independent repeat profiles in the case of oxytocin and angiotensin IV, respectively (thin transparent lines). (b) Representative snapshots were taken from umbrella windows at a particular value of the reaction coordinate. The R and TI snapshots were taken from 2.7 Å and 1.5 Å for both peptides while the TS snapshots were taken from 1.8 Å and 1.7 Å for oxytocin and angiotensin IV, respectively. The Zn^{2+} ion is shown as gray sphere. Only important side chains were shown while the rest of the active site residues and non-polar hydrogens weas omitted for clarity. All PMFs have been calculated at 310 K.

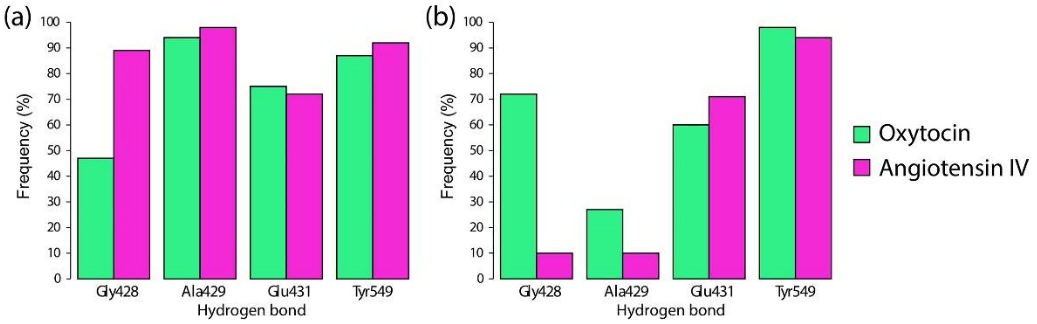

Figure S12 The frequency of hydrogen bonds between oxytocin and angiotensin IV and key residues in the active site of IRAP extracted from QM/MM MD umbrella sampling simulations of (a) reactant and (b) tetrahedral intermediate.

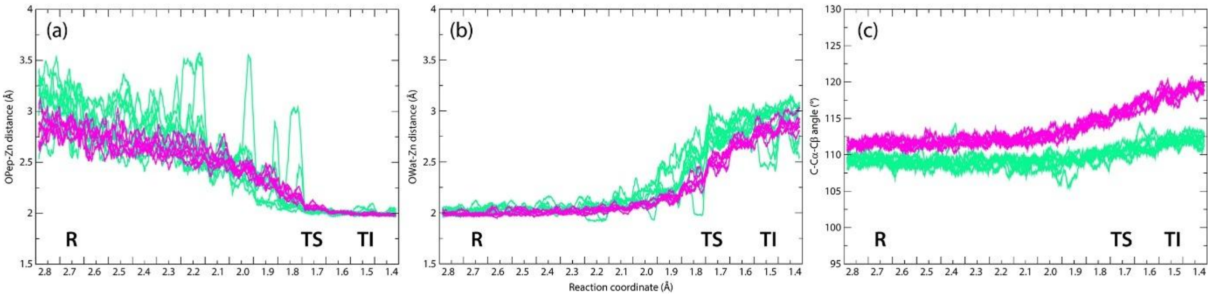

Figure S13 Evolution of (a) distance between scissile peptide oxygen and zinc, (b) distance between catalytic water oxygen and zinc, and (c) angle between C-Cα-Cβ atoms along the tetrahedral intermediate (TI) formation reaction in oxytocin and angiotensin IV.

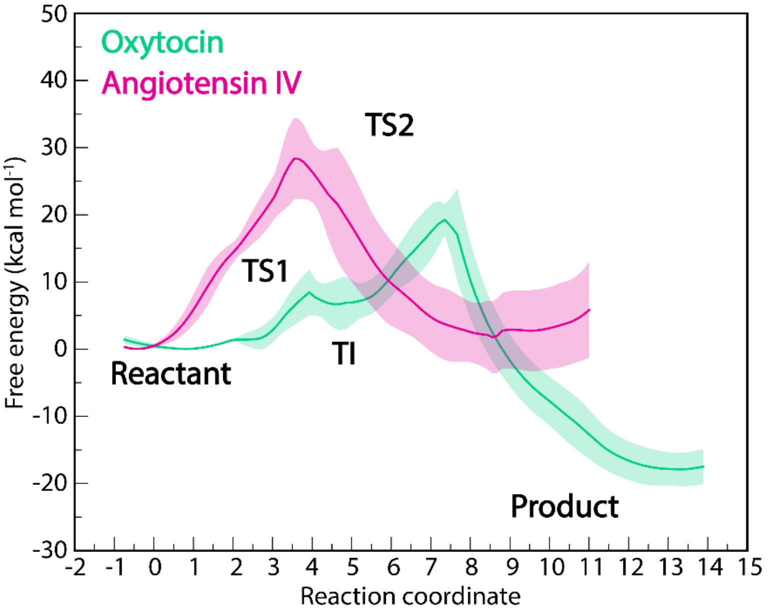

Figure S14 Average potential of mean force (PMF) calculated along the minimum free energy path (MFEP) for the N-terminal peptide bond cleavage in oxytocin (green) and angiotensin IV (magenta) obtained from adaptive string QM/MM MD simulations starting from Glu465 in OE2 conformation. Average free energy profiles (thick lines) were calculated from three repeat profiles with the errors shown as transparent shaded regions. All PMFs have been calculated at 300 K. The reaction coordinate is given in a.m.u.$^{1/2}$Å.

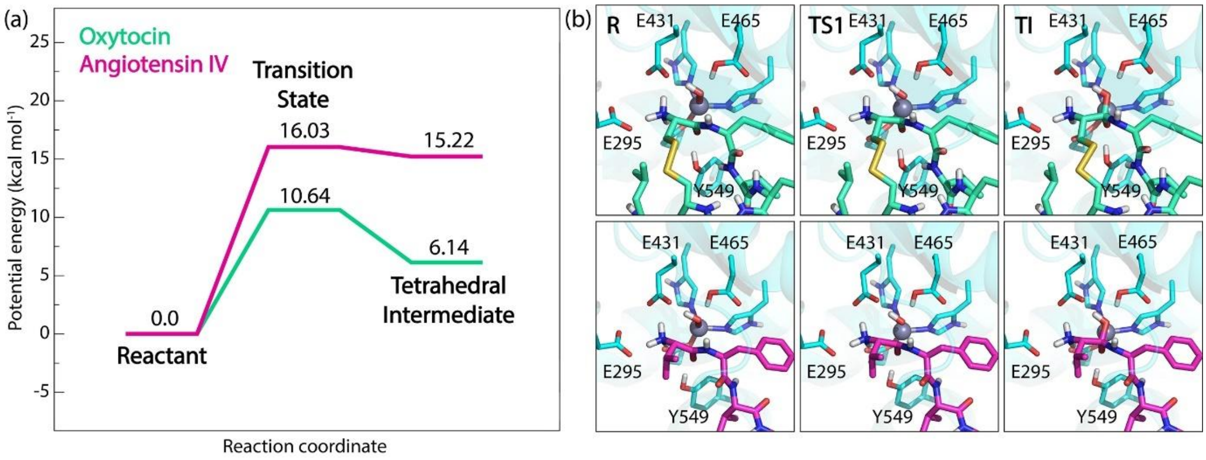

Figure S15 (a) Potential energy profile for the IRAP-catalyzed nucleophilic attack of hydroxide on the carbonyl carbon of the scissile peptide bond forming the oxyanion tetrahedral intermediate in oxytocin (green) and angiotensin IV (magenta) obtained from QM/MM calculations performed at the B3LYP-D3BJ/def2-TZVP:AMBER//B3LYP-D3BJ/6-31G(d):AMBER level of theory. (b) Optimized geometries of the reactant, transition state and tetrahedral intermediate for the reaction between IRAP and oxytocin and angiotensin IV. The Zn^{2+} ion is shown as gray sphere. Only important sidechains and polar hydrogens are shown for clarity.

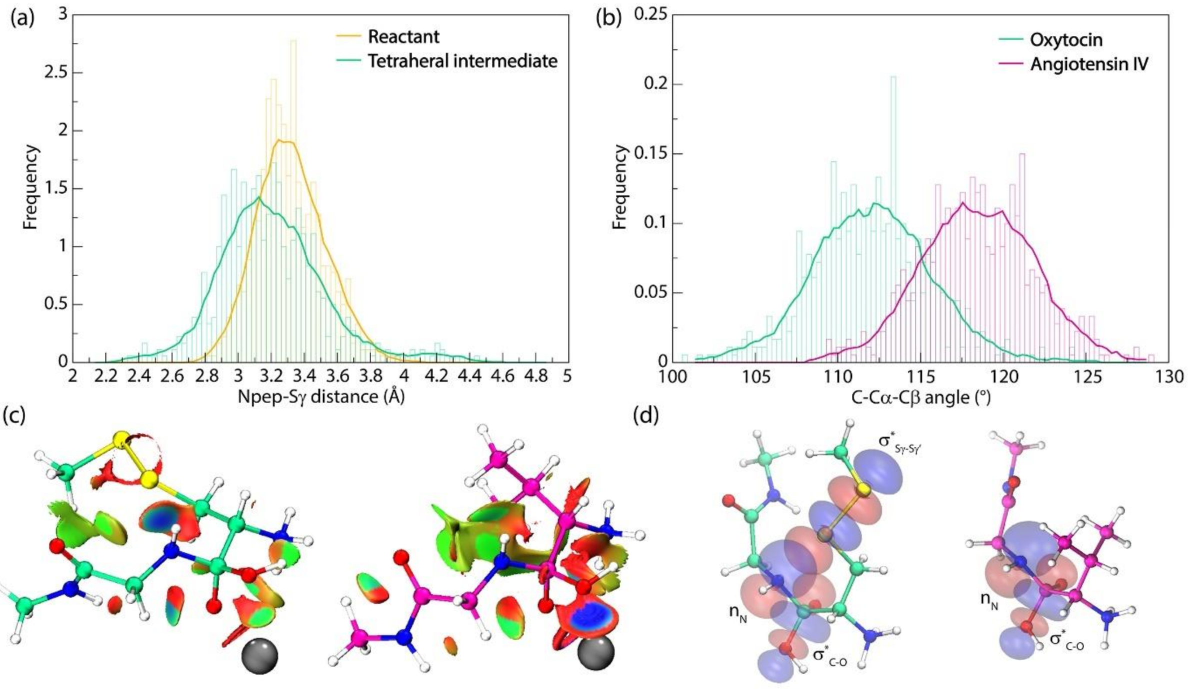

Figure S16 (a) Histograms of distance between the scissile peptide nitrogen atom and Sγ of the N-terminal cysteine calculated from DFTB3/MM umbrella simulations at 2.7 Å for the reactant and 1.5 Å for the tetrahedral intermediate along the reaction with oxytocin. (b) Histograms of angle between C-Cα-Cβ of the N-terminal cysteine and valine calculated from DFTB3/MM umbrella simulations at 1.5 Å for the TI in oxytocin and angiotensin IV. (c) Non-covalent interaction (NCI) analysis indicating type of interaction in the TI state obtained by performing a gas phase B3LYP-D3BJ/def2-TZVP single point calculation on the isolated peptide with Zn^{2+} taken from the full QM/MM optimized structures. The isosurfaces are colored with a blue-green-red scale according to the values of $sign(\lambda_2)\rho$, ranging from -0.035 to 0.02 a.u. Blue indicates strong attractive and red indicates strong repulsive interaction. Green indicates weak attractive Van der Waals forces. The isovalue is set to 0.7. (d) Stabilizing NBOs in the TI obtained using second order perturbation theory analysis of Fock matrix from gas phase B3LYP-D3BJ/def2-TZVP single point calculation of the full QM region taken from the QM/MM optimized structures. Only NBOs involving hydroxide and peptide are shown for clarity. The isovalue of 0.05 was used.

Table S1 Selected bonds, angles and dihedrals calculated using QM/MM optimized geometries describing the TI formation in oxytocin (upper) and angiotensin IV (lower). The schematic representation of calculated geometrical parameters is shown below the table for both peptides.

**Oxytocin**	**R**	**TS**	**TI**
	Bond (Å)		
Owat-Cpep	2.58	1.91	1.45
Cpep-Opep	1.24	1.29	1.37
Owat-Zn	1.93	2.10	2.90
Opep-Zn	2.57	2.12	1.93
Npep-Sγ	3.31	3.20	2.72
	Angle (°)		
Owat-Cpep-Opep	83.40	95.02	109.01
Owat-Zn-Opep	73.12	69.22	52.18
C-Cα-Cβ	107.29	109.14	112.72
	Dihedral (°)		
N-Cα-C-N	162.22	173.94	173.70
**Angiotensin IV**	**R**	**TS**	**TI**
	Bond (Å)		
Owat-Cpep	2.60	1.83	1.51
Cpep-Opep	1.24	1.30	1.36
Owat-Zn	1.90	2.05	2.32
Opep-Zn	2.99	2.16	1.99
	Angle (°)		
Owat-Cpep-Opep	92.20	97.74	103.69
Owat-Zn-Opep	69.51	68.94	62.52
C-Cα-Cβ	110.49	114.84	118.15
	Dihedral (°)		
N-Cα-C-N	159.78	174.52	163.68

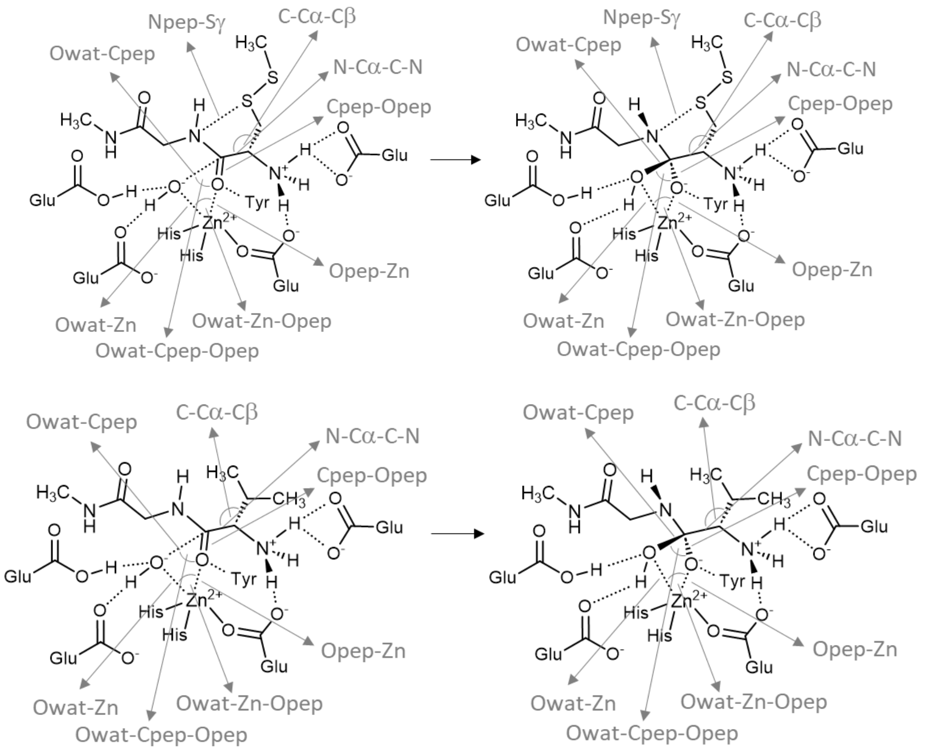

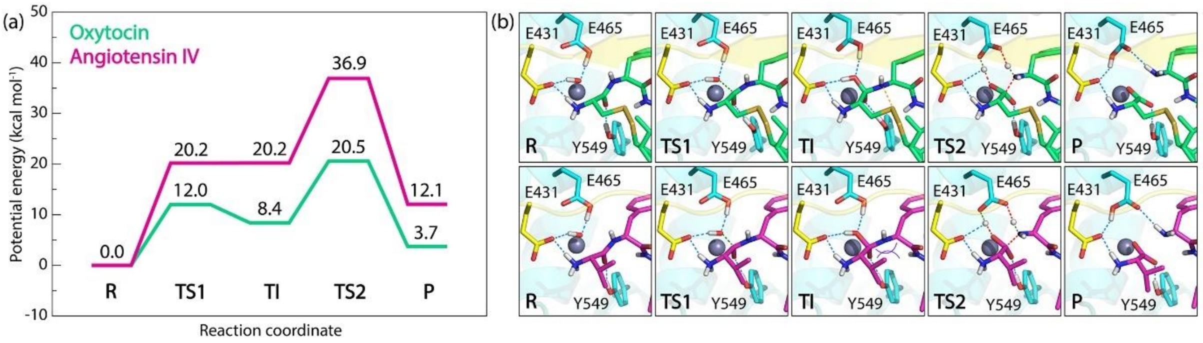

Figure S17 (a) Potential energy profile for the IRAP-catalyzed nucleophilic attack of hydroxide on the carbonyl carbon of the scissile peptide bond forming the oxyanion tetrahedral intermediate and the peptide bond cleavage in oxytocin (green) and angiotensin IV (magenta) obtained from QM/MM calculations performed at the B3LYP-D3/def2-TZVP:AMBER//B3LYP-D3/6-31G(d):AMBER level of theory starting from Glu465 in OE2 conformation. (b) Optimized geometries of the reactant, transition state, intermediate and product for the reaction between IRAP and oxytocin and angiotensin IV. The Zn^{2+} ion is shown as gray sphere. Only important sidechains and polar hydrogens are shown for clarity.

Table S2 Summary of all calculated energies (kcal mol^{-1}) for N-terminus peptide bond cleavage in oxytocin and angiotensin IV catalyzed by IRAP.

	DFTB3/MM				DFT/MM			
	Umbrella sampling		Adaptive string method		First step		Both steps	
	Oxt	Ang IV	Oxt	Ang IV	Oxt	Ang IV	Oxt	Ang IV
R	0.0	0.0	0.0	0.0	0.0	0.0	0.0	0.0
TS1	9.0	14.5	8.0	14.0	10.6	16.0	12.0	20.2
TI	7.0	14.0	5.5	13.5	6.1	15.2	8.4	20.2
TS2			21.5	27.6			20.5	36.9
P			-16.5	-4.0			3.7	12.1

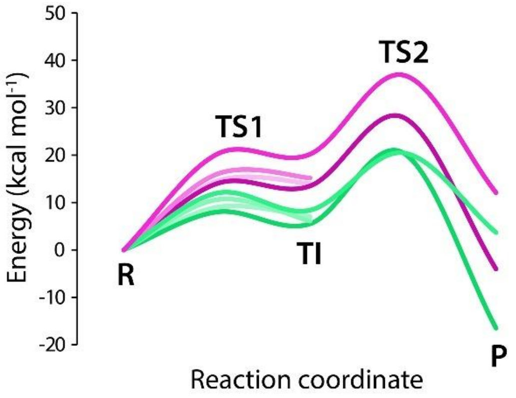

Figure S18 Summary of all calculated energies for N-terminus peptide bond cleavage in oxytocin and angiotensin IV catalyzed by IRAP.

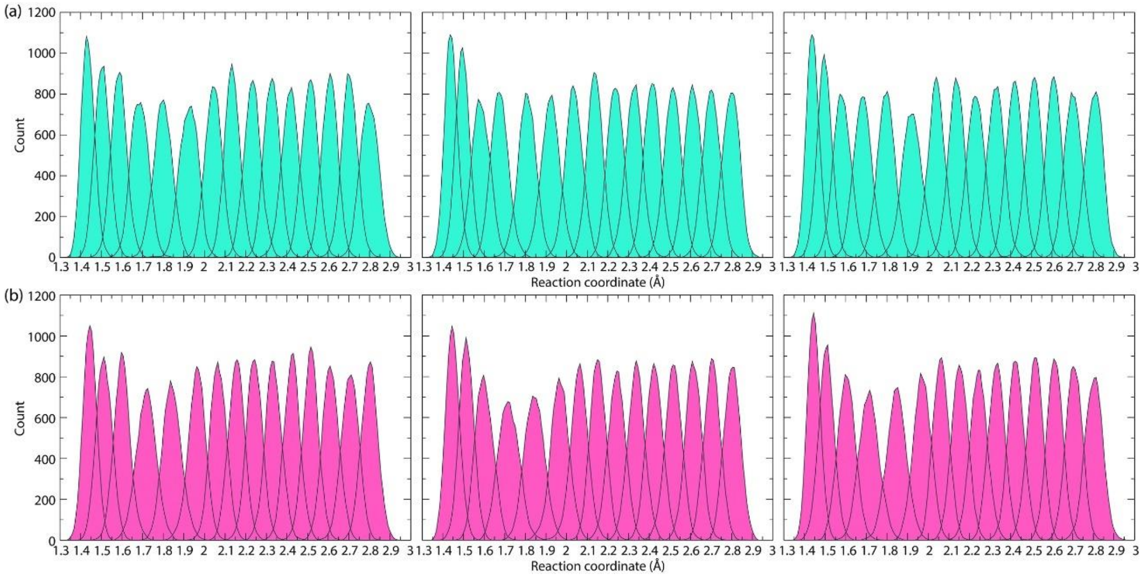

Figure S19 Histograms showing the overlap between neighboring windows along the reaction coordinate (O_{Water}-$C_{Peptide}$ distance) for the Glu465-catalyzed (OE1 conformation) nucleophilic attack of oxygen from water on the carbonyl carbon of the scissile peptide bond in oxytocin (green) and angiotensin IV (magenta) obtained from umbrella sampling QM/MM MD simulations.

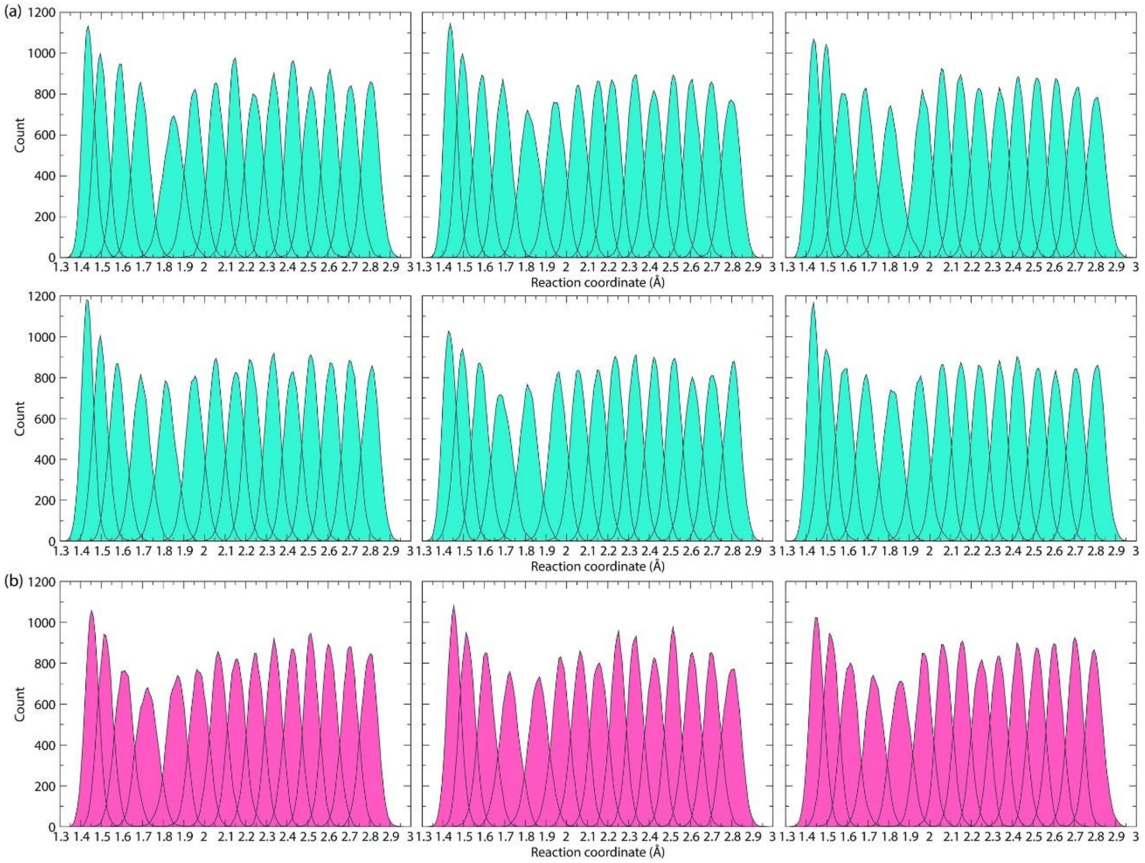

Figure S20 Histograms showing the overlap between neighboring windows along the reaction coordinate (O_{Water}-$C_{Peptide}$ distance) for the Glu465-catalyzed (OE2 conformation) nucleophilic attack of oxygen from water on the carbonyl carbon of the scissile peptide bond in oxytocin (green) and angiotensin IV (magenta) obtained from umbrella sampling QM/MM MD simulations.

References

[1] Wong, A. H.; Zhou, D.; Rini, J. M. The X-ray crystal structure of human aminopeptidase N reveals a novel dimer and the basis for peptide processing. *J. Biol. Chem.* **2012**, *287*, 36804–36813.
[2] Jumper, J. M.; et al. Accurate structure prediction of biomolecular interactions with AlphaFold 3. *Nature* **2024**, *630*, 493–500.
[3] Case, D. A.; Ben-Shalom, I. Y.; Brozell, S. R.; Cerutti, D. S.; Cheatham III, T. E.; Cruzeiro, V. W. D.; Darden, T. A.; Duke, R. E.; Ghoreishi, D.; Gilson, M. K.; Gohlke, H.; Goetz, A. W.; Greene, D.; Harris, R.; Homeyer, N.; Izadi, S.; Kovalenko, A.; Kurtzman, T.; Lee, T. S.; LeGrand, S.; Li, P.; Lin, C.; Liu, J.; Luchko, T.; Luo, R.; Mermelstein, D. J.; Merz, K. M.; Miao, Y.; Monard, G.; Nguyen, C.; Nguyen, H.; Omelyan, I.; Onufriev, A.; Pan, F.; Qi, R.; Roe, D. R.; Roitberg, A.; Sagui, C.; Schott-Verdugo, S.; Shen, J.; Simmerling, C. L.; Smith, J.; Salomon-Ferrer, R.; Swails, J.; Walker, R. C.; Wang, J.; Wei, H.; Wolf, R. M.; Wu, X.; Xiao, L.; York, D. M.; Kollman, P. A. (2018), AMBER 2018. University of California, San Francisco.
[4] Faller, R.; de Pablo J. J. Constant pressure hybrid Molecular Dynamics–Monte Carlo simulations. *Chem. Phys.* **2002**, *116*, 55–59.
[5] The PyMOL Molecular Graphics System, Version 3.1 Schrödinger, LLC.
[6] Humphrey, W.; Dalke, A; Schulten, K. VMD - Visual Molecular Dynamics. *J. Molec. Graphics.* **1996**, *14*, 33–38.
[7] Contreras-García, J.; Johnson, E. R.; Keinan, S.; Chaudret, R.; Piquemal, J. P.; Beratan, D. N.; Yang, W. NCIPLOT: a program for plotting non-covalent interaction regions. *J. Chem. Theory Comput.* **2011**, *7*, 625–632.
[8] Lu, T.; Chen, Q. Interaction Region Indicator: A Simple Real Space Function Clearly Revealing Both Chemical Bonds and Weak Interactions. *Chemistry Methods* **2021**, *1*, 231–239.
[9] Lu, T.; Chen, F. Multiwfn: A Multifunctional Wavefunction Analyzer. *J. Comput. Chem.* **2012**, *33*, 580–592.
[10] Frisch, M. J.; Trucks, G. W.; Schlegel, H. B.; Scuseria, G. E.; Robb, M. A.; Cheeseman, J. R.; Scalmani, G.; Barone, V.; Mennucci, B.; Petersson, G. A.; Nakatsuji, H.; Caricato, M.; Li, X.; Hratchian, H. P.; Izmaylov, A. F.; Bloino, J.; Zheng, G.; Sonnenberg, J. L.; Hada, M.; Ehara, M.; Toyota, K.; Fukuda, R.; Hasegawa, J.; Ishida, M.; Nakajima, T.; Honda, Y.; Kitao, O.; Nakai, H.; Vreven, T.; Montgomery Jr., J. A.; Peralta, J. E.; Ogliaro, F.; Bearpark, M.; Heyd, J. J.; Brothers, E.; Kudin, K. N.; Staroverov, V. N.; Kobayashi, R.; Normand, J.; Raghavachari, K.; Rendell, A.; Burant, J. C.; Iyengar, S. S.; Tomasi, J.; Cossi, M.; Rega, N.; Millam, J. M.; Klene, M.; Knox, J. E.; Cross, J. B.; Bakken, V.; Adamo, C.; Jaramillo, J.; Gomperts, R.; Stratmann, R. E.; Yazyev, O.; Austin, A. J.; Cammi, R.; Pomelli, C.; Ochterski, J. W.; Martin, R. L.; Morokuma, K.; Zakrzewski, V. G.; Voth, G. A.; Salvador, P.; Dannenberg, J. J.; Dapprich, S.; Daniels, A. D.; Farkas, O.; Foresman, J. B.; Ortiz, J. V; Cioslowski, J.; Fox, D. J. Gaussian16 (Revision A.03), Gaussian Inc. Wallingford CT. 2016.
[11] Lu, X.; Fang, D.; Ito, S.; Okamoto, Y.; Ovchinnikov, V.; Cui, Q. QM/MM Free Energy Simulations: Recent Pro-gress and Challenges. *Mol. Simul.* **2016**, *42*, 1056–1078.
[12] Grossfield, A. "WHAM: the weighted histogram analysis method", version 2.0.11, http://membrane.urmc.rochester.edu/wordpress/?page_id=126
[13] Grimme, S.; Ehrlich, S.; Goerigk, L. Effect of the damping function in dispersion corrected density functional theory. *J. Comput. Chem.* **2011**, *32*, 1456–1465.
[14] Grimme, S.; Hansen, A.; Brandenburg, J. G.; Bannwarth, C. Dispersion-Corrected Mean-Field Electronic Structure Methods. *Chem. Rev.* **2016**, *116*, 5105–5154.
[15] Lu, Y.; Sen, K.; Yong, C.; Gunn, D. S. D.; Purton, J. A.; Guan, J.; Desmoutier, A.; Abdul Nasir, J.; Zhang, X.; Zhu, L.; Hou, Q.; Jackson-Masters, J.; Watts, S.; Hanson, R.; Thomas, H. N.; Jayawardena, O.; Logsdail, A. J.; Woodley, S. M.; Senn, H. M.; Sherwood, P.; Catlow, R. A.; Sokol, A. A.; Keal, T. W. Multiscale QM/MM modelling of catalytic systems with ChemShell. *Phys. Chem. Chem. Phys.* **2023**, *25*, 21816–21835.
[16] Lu, Y.; Farrow, M. R.; Fayon, P.; Logsdail, A. J.; Sokol, A. A.; Catlow, C. R. A.; Sherwood, P.; Keal, T. W. Open-Source, Python-Based Redevelopment of the ChemShell Multiscale QM/MM Environment. *J. Chem. Theory Comput.* **2019**, *15*, 1317–1328.
[17] Foster, J. P.; Weinhold, F. Natural hybrid orbitals. *J. Am. Chem. Soc.* **1980**, *102*, 7211–7218.
[18] Li, G. B.; Abboud, M. I.; Brem, J.; Someya, H.; Lohans, C. T.; Yang, S. Y.; Spencer, J.; Wareham, D. W.; McDonough, M. A.; Schofield, C. J. NMR-filtered virtual screening leads to non-metal chelating metallo-β-lactamase inhibitors. *Chem Sci.* **2017**, *8*, 928–937.
